# Malaria: past, present, and future

**DOI:** 10.1038/s41392-025-02246-3

**Published:** 2025-06-17

**Authors:** Qilong Li, Tong Liu, Kunying Lv, Fulong Liao, Jigang Wang, Youyou Tu, Qijun Chen

**Affiliations:** 1 Key Laboratory of Livestock Infectious Diseases, Ministry of Education, and Key Laboratory of Ruminant Infectious Disease Prevention and Control (East), Ministry of Agriculture and Rural Affairs, College of Animal Science and Veterinary Medicine, Shenyang Agricultural University, Shenyang, China; 2 Research Unit for Pathogenic Mechanisms of Zoonotic Parasites, Chinese Academy of Medical Sciences, Shenyang, China; 3 Artemisinin Research Center, and Institute of Chinese Materia Medica, China Academy of Chinese Medical Sciences, Beijing, China

**Keywords:** Infectious diseases, Microbiology

## Abstract

Malaria, caused by *Plasmodium* parasites and transmitted by *Anopheles* mosquitoes, greatly impacts public health and socioeconomic development, particularly in sub-Saharan African countries. Despite advances in malaria treatment and prevention, the number of clinical cases and deaths have increased in recent years. The complex life cycle and genetic diversity of *Plasmodium* parasites pose significant challenges in drug and vaccine development, particularly due to the emerging partial resistance of parasites to artemisinin. With the availability and application of state-of-the-art biotechnology in recent years, knowledge in terms of parasite biology, pathogenicity, host–parasite interactions and pathogenesis has advanced tremendously. This review highlights the most recent research progress and understanding in *Plasmodium* biology, with a primary focus on *P. falciparum* and associated pathogenesis. The therapeutic targets and progress in the clinical application of anti-malaria drugs have also been summarized. The FDA-approved regimens like Artemether-Lumefantrine, Atovaquone-Proguanil, and Primaquine are discussed, and their benefits and limitations are highlighted, especially in terms of drug resistance. Perspectives in the development of novel vaccines and new drugs, such as Sevuparin, Imatinib, and Cipargamin, and combination therapies with promise in overcoming resistance has been proposed. Overall, this review provides a detailed summary of the latest progress in malaria research and emphasizes the need for continuous monitoring and innovation in malaria treatment.

## Introduction

Malaria, a disease caused by several *Plasmodium* species, namely *P. falciparum*, *P. vivax*, *P. malariae*, *P. ovale* and *P. knowlesi*, profoundly impacts human health.^1–6^ Mixed infections of *P. falciparum* with *P. malariae* and/or *P. ovale* in Africa^7^ and *P. falciparum with P. vivax* and/or *P. knowlesi* in Southeast Asia have posed challenges in disease control.^8,9^ In contrast to earlier optimistic predictions of malaria eradication by 2030, the number of cases has been increasing in recent years.^10^ According to the latest WHO’s World malaria report, global malaria cases in 2023 continually increased. An estimated 263 million cases occurred in 83 malaria-endemic countries, with Nigeria (25.9%), the Democratic Republic of the Congo (12.6%), Uganda (4.8%), Ethiopia (3.6%) and Mozambique (3.5%) accounting for over half of all cases. Malaria also leads to great economic losses of approximately $12 billion US dollars per year in sub-Saharan Africa.^11^

Once injected into the human host by the female *Anopheles* mosquito, the sporozoites travel to the liver for differentiation.^8,12^ After multiplication and adaptation in hepatocytes for 6–7 days, thousands of merozoites egress from hepatic cells to infect red blood cells (RBCs).^13^ A recent study revealed that *P. falciparum* transmission is disproportionately driven by infected school-aged boys who receive a high number of mosquito bites, with infectious mosquitoes preferentially biting already infected individuals, highlighting the importance of targeted interventions for interrupting malaria transmission.^14^ The invasion and intracellular development of malaria parasites in RBCs results in various pathologies in the host, with clinical symptoms including periodic fever episodes (a cyclical occurrence of sudden coldness followed by shivering and then fever and sweating), headache, chills, and vomiting.^15^ Without prompt treatment, *P. falciparum* malaria can progress to severe illness and death, with symptoms such as severe anemia, respiratory distress, or cerebral malaria (CM).^16^

Malaria treatment regimens are based on the parasite type, symptom severity, and patient age.^17^ Classical antimalarial drugs such as chloroquine, quinine, pyronaridine, pyrimethamine, primaquine, and piperaquine have been widely applied in clinics to for decades. However, with the emergence of classical antimalarial drug resistance, especially in *P. falciparum*, artemisinin (ART)-based combination therapy (ACT) has been recommended as the first-line treatment. In this respect, Chinese scientists have made important contributions. Professor Tu Youyou was the pioneer who discovered a rational method for extracting the active ingredient, artemisinin, from the *Artemisia annua* plant and conducted the first clinical trial in patients.^18^ Professors Zhou Weishan and Mrs. Luo Zeyuan resolved the structural determination and synthesized the structures of artemisinin.^18^ Later, Professor Li Guoqiao’s team developed the ACT regimen with an aim to overcome resistance to single-drug treatments.^18^ However, ART-resistant strains of *P. falciparum* have now been frequently detected in African and Southeast Asian countries, presenting a great challenge for disease control.^19^ The underlying mechanisms of the emergence of drug-resistant *P. falciparum* have attracted tremendous attention, and gene mutations and duplication have been regarded as the main causes, whereas the specific mechanism of ART resistance is a debated issue that will be discussed later.

This review will explore various aspects of malaria, starting with its epidemiology, where will examine the global distribution and prevalence of the disease, as well as trends in malaria incidence and mortality. Next, this review will delve into parasite genomics, providing an overview of *Plasmodium* species genotypes and discussing recent advances in genomic research, including the use of single-cell RNA sequencing. The clinical features of malaria will be discussed in detail, focusing on symptoms such as fever, cerebral, and placenta-associated malaria. We will then explore signaling pathways and crosstalk, highlighting the regulatory mechanisms that govern parasite development and invasion, as well as key pathways involved in *Plasmodium* development. The pathogenic mechanisms will be addressed next, with a focus on the molecular mechanisms of host cell recognition and cytoadherence, along with the immune response strategies employed by the parasites. Finally, the review will conclude with an overview of therapeutic targets and clinical research progress, covering the challenges of drug resistance, emerging therapeutic targets, and recent developments in malaria treatment, including promising clinical trials and FDA-approved drugs. This review will provide a comprehensive understanding of the current state of malaria research and its future

### The research history and milestone events in studies on malaria

**Fig. 1:**
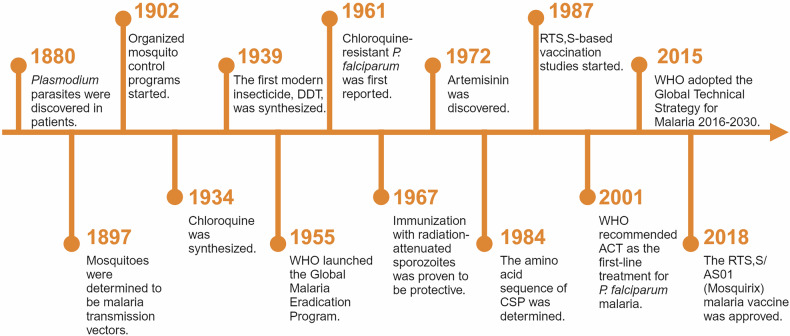
Milestones in malaria. The key milestones in the history of malaria research and control are depicted in this timeline. It highlights major discoveries, the development of treatments and vaccines, and significant global initiatives from the identification of *Plasmodium* parasites in 1880 to the approval of the RTS, S/AS01 (Mosquirix) malaria vaccine in 2018. This figure was created with BioRender.com

Malaria research has evolved through a series of landmark discoveries and milestone events, each contributing to our understanding of the disease and its control (Fig. 1). The modern study of malaria began in 1880, when the French military doctor Charles Louis Alphonse Laveran discovered *Plasmodium* parasites in the blood of infected patients,^20^ earning him the 1907 Nobel Prize in Physiology or Medicine.^21^ This established malaria as a parasitic disease, laying the foundation for further exploration.^22^ In 1897, British physician Sir Ronald Ross demonstrated that Anopheles mosquitoes are the vectors of malaria and elucidated the developmental stages of *Plasmodium* in the mosquito.^23^ For this pivotal work, Ross was awarded the 1902 Nobel Prize in Physiology or Medicine. These findings revolutionized malaria control efforts, enabling vector management strategies that remain integral to modern malaria prevention.^24^ The 20th century saw several groundbreaking advancements. In 1927, the Austrian psychiatrist Julius Wagner-Jauregg received the Nobel Prize in Physiology or Medicine for his innovative use of *Plasmodium* infection to treat neurosyphilis-induced paralysis.^25^ While controversial, this method highlighted malaria’s potential for therapeutic applications in a pre-antibiotic era.^26,27^ In 1965, American chemist Robert Burns Woodward was awarded the Nobel Prize in Chemistry for the first total synthesis of quinine,^28,29^ one of the earliest and most effective antimalarial drugs.^30,31^ His work underscored the role of chemistry in developing treatments for malaria and other diseases.^32,33^ The mid-20th century was also marked by the introduction of synthetic antimalarials such as chloroquine (1934)^34^ and the first modern insecticide Dichloro-diphenyl-trichloroethane (DDT, 1939),^35^ which became central to the World Health Organization’s (WHO) global malaria eradication campaign launched in 1955.^36^ Despite early successes, the emergence of resistance to both chloroquine and DDT revealed the need for sustained innovation and comprehensive strategies.^37,38^

A major breakthrough came in 1972, when Chinese scientist Tu Youyou discovered a simple technique and extracted the potent anti-malaria component, artemisinin,^39^ from the traditional Chinese medicinal plant *Artemisia annua*.^18^ She was awarded the Nobel Prize in Physiology or Medicine for the astonishing discovery in 2015. Artemisinin and its derivatives form the basis of ACT, which are currently the most adapted regimen for treating drug-resistant *Plasmodium falciparum*.^40,41^ Her work, inspired by ancient Chinese medical texts and validated through modern pharmacological research, has saved millions of lives and remains a cornerstone of global malaria treatment. In the year 2000, the WHO launched the Global Technical Strategy for Malaria 2016–2030,^42^ setting ambitious goals to reduce malaria incidence and mortality rates by at least 90% by 2030 compared to 2015 levels. This goal is unlikely to be achieved, due to the fact that the 2023 global malaria incidence was nearly three times higher than that of the WHO’s aim. In the meantime, malaria vaccines have been pursued by various approaches including attenuation of the sporozoites by radiation.^43^ Experiments in rodent models propelled scientists to identify the “target antigen” on the sporozoite surface, which led to the cloning of the gene coding for the circumsporozoite surface protein (CSP) in *Pf* malaria.^44–46^ CSP has, since then, been regarded as the primary malaria vaccine candidate.^47^ Later, the central repetitive region of CSP was selected and biosynthesized (expressed) in a fused form with the S-antigen of the Hepatitis B virus. The product of the recombinant fusion protein was named RTS,S.^48^ After several rounds of clinical trials in African adults and children, RTS,S/AS01_E_ (Mosquirix) became the first malaria vaccine approved by the WHO,^49^ representing a milestone in prevention strategies.^50,51^ The vaccine’s pilot implementation in sub-Saharan Africa offered hope for reducing the disease burden in high-risk populations.^52^ The completion of the genome sequencing of several *Plasmodium* species marked a new era in research on malaria, which has provided innovative pathways accelerating the process of both drug mining and vaccine development.^53,54^ From 2016 to 2024, several countries achieved malaria-free certification by WHO, including Algeria and Argentina in 2019,^55^ China in 2021,^56^ Azerbaijan and Tajikistan in 2023, and Belize and Egypt in 2024, showcasing the success of elimination campaigns. Another major development occurred in 2024 with the WHO approval of the R21/Matrix-M malaria vaccine, which meets the efficacy target of 75% in young African children.^57^

### Epidemiology of malaria

Malaria remains a significant global health challenge, with an estimated 263 million cases reported in 83 endemic countries across five WHO regions in 2023, reflecting a slight increase from 11 million cases in 2022, according to WHO’s World Malaria Report 2024 (www.who.int/teams/global-malaria-programme). Of the 93 countries that were malaria endemic in 2015, 26% (including those that are now certified malaria free) met the GTS morbidity milestone for 2023, 34% made progress in reducing malaria case incidence but by less than the expected target, 15% had similar incidence to 2015 and 26% experienced an increase in case incidence. Despite some progress in malaria control, several factors, including funding gaps, poverty, and climate change, have contributed to setbacks in global efforts to reduce malaria transmission.

Sub-Saharan Africa remains the region most affected by malaria, accounting for ~94% of global cases in 2023, with the highest burden concentrated in countries such as Nigeria (30.9%), the Democratic Republic of the Congo (11.3%), Niger (5.9%), and United Republic of Tanzania (4.3%). In 2023, the region reported 246 million cases and 569,000 deaths. The overwhelming prevalence of *P. falciparum*, the most virulent malaria species, exacerbates the disease burden, especially among vulnerable groups such as young children^58^ and pregnant women.^59^ While adults in endemic areas often develop partial immunity,^60^ young children continue to face the greatest risk of severe disease.^61^ The high transmission rates are largely driven by favorable environmental factors, including the tropical climate, which supports year-round breeding of *Anopheles* mosquitoes.^62–64^ Despite this, significant challenges persist in controlling malaria, such as weak health infrastructure, limited access to diagnostic tools, and the high cost of prevention measures (such as insecticide-treated bed nets and antimalarial medications).^65^ These barriers hinder the effectiveness of malaria control efforts and contribute to the ongoing high burden of the disease. The rapid spread of artemisinin partial resistance (ART-R) in Africa also poses a serious threat to malaria control efforts, with potential economic and health impacts. Urgent regional initiatives are required to address ART-R through coordinated cross-border actions, enhanced surveillance, diversified treatments, and strengthened health systems, similar to the successful approaches in Southeast Asia, to prevent the further spread of resistance and safeguard malaria elimination goals.^66^

South-East Asia exhibits a mixed malaria burden, reporting 4 million cases in 2023 according to World Malaria Report 2024. While some countries, such as India (51%), Indonesia (27%), and Myanmar (21%), experience high transmission rates, others, such as Viet Nam (only 370 cases), have made considerable strides in malaria elimination. India remains one of the largest contributors to malaria cases in the region, reporting 48% of all cases in the region were due to *P. vivax*. A major concern for Asia is the growing problem of drug resistance, particularly to ART. Resistance has been detected in several countries, including Cambodia, Thailand, Myanmar, and Vietnam, raising alarm about the future effectiveness of ACTs.^67^ In recent years, *P. knowlesi* infection has become an increasingly significant issue in malaria cases, particularly in Southeast Asia, with prevalence most prominent in Indonesia, Malaysia, and Thailand, and more recently observed in Cambodia. On a global scale, 3,290 cases of *P. knowlesi* infection were documented in 2023, reflecting an 18.9% rise from the 2768 cases reported in 2022. Similarly, indigenous cases of *P. knowlesi* showed a 22% increase, growing from 2682 in 2022 to 3274 in 2023.

Malaria in the Americas is primarily confined to Brazil (33%), the Bolivarian Republic of Venezuela (26%), Colombia (21%), Guyana (6%), and Peru (4%) reporting the highest burden according to World Malaria Report 2024. In 2023, the region recorded ~505,642 cases. All indigenous malaria cases reported by Guatemala and Mexico were attributed to *P. vivax*. In the Bolivarian Republic of Venezuela, Brazil, Colombia, Ecuador, French Guiana, Guyana, Honduras, Nicaragua, Panama, Peru, and the Plurinational State of Bolivia, *P. vivax* accounted for 60% to 99% of the documented indigenous cases. Conversely, all indigenous cases reported by the Dominican Republic and Haiti, along with 92% of the indigenous cases recorded in Costa Rica in 2023, were attributed to *P. falciparum*. Colombia reported the highest number of *P. falciparum* cases in the region. Although malaria transmission is less intense than that in sub-Saharan Africa, challenges remain in remote and rural areas where healthcare access is limited, and migratory movements increase the risk of malaria transmission. Efforts to control malaria in South America include interventions such as indoor residual spraying and mass drug administration (MDA) programs. However, regional differences in program effectiveness highlight the need for tailored approaches. Resistance to chloroquine, the traditional first-line treatment for *P. vivax*, remains a concern in certain areas, further complicating control efforts.

Malaria transmission in Western Pacific Region is mainly concentrated in Papua New Guinea (88%), which continues to experience a high burden of both *P. falciparum* (71%) and *P. vivax* (29%) according to World Malaria Report 2024. In 2023, the region recorded an estimated 1.7 million malaria cases and 3360 deaths. This represents a 5% increase in cases and a 3% reduction in deaths compared to 2010. Papua New Guinea remains one of the few countries outside sub-Saharan Africa with significant malaria transmission, with the disease contributing to considerable morbidity. In contrast, the Pacific islands have largely succeeded in eliminating indigenous malaria transmission, with countries such as Australia, New Zealand, and several island nations achieving malaria-free status.

Malaria cases in the WHO Eastern Mediterranean Region were estimated to have decreased by 37.7% between 2000 and 2015, dropping from 6.9 million to 4.3 million according to World Malaria Report 2024. However, this trend reversed, with cases rising by 137% between 2015 and 2023, reaching an estimated 10.2 million. Notably, there was a significant increase of 62% between 2021 and 2023, largely driven by a malaria outbreak in Pakistan, which saw a rise of 3.7 million cases following catastrophic flooding that affected over 30 million people. Several countries experienced notable increases in malaria cases, with Afghanistan seeing a rise in estimated cases from 288,000 in 2022 to 424,000 in 2023. In the same year, *P. vivax* accounted for 35.2% of the cases in the region, primarily in Afghanistan and Pakistan. However, due to ongoing instability and significant security challenges in Sudan, as well as incomplete reporting in Yemen, comprehensive data collection remains a challenge. As a result, recent estimates of malaria burden in these countries should be interpreted with caution. To address this, WHO is supporting subnational burden estimation efforts in these nations to improve decision-making and guide malaria control strategies in regions with unstable conditions.

In addition to the regional trends, several global challenges have complicated efforts to control malaria. The World Malaria Report 2024 highlights the substantial risk posed by climate change, which can alter the behavior of malaria vectors and increase the areas at risk of transmission. Extreme weather events, such as floods^68^ and heatwaves,^69^ have been linked to increased malaria outbreaks, though the precise relationship between climate change and malaria transmission remains unclear. The COVID-19 pandemic has also significantly disrupted malaria control efforts,^70,71^ leading to delays in the distribution of mosquito nets,^72^ diagnostic tools,^73,74^ and antimalarial treatments.^75^ Many countries reported a decline in malaria-related services, exacerbating the disease burden in already high-risk areas.

Despite some notable progress in malaria control and the introduction of new interventions, including the RTS, S/AS01_E_ malaria vaccine and the recommendation of the R21/Matrix-M vaccine, the global malaria burden remains high. The increase in malaria cases in 2024 compared to pre-pandemic levels underscores the ongoing need for comprehensive and sustained malaria control efforts. The emergence of drug resistance, climate change, and the lingering effects of the COVID-19 pandemic present significant challenges and addressing these issues will be crucial for meeting the global malaria elimination targets. Continued investment in research, surveillance, and the development of innovative tools and strategies is essential for reducing the global burden of malaria and ultimately achieving its eradication.

### Plasmodium genomics

Ancestors of the *Plasmodium* parasite clade may have been free-living protozoa with chloroplasts that adapted to living in the intestines of aquatic invertebrates.^76^ The evolution of *Plasmodium* species involved a shift from an ancestor that performed photosynthesis to a complex parasite with a crucial apicoplast for host adaptation.^77^ DNA sequence comparisons suggest that the origins of *Plasmodium* parasites are closely linked to their hosts.^78–80^ This is supported by a comprehensive analysis of the mitochondrial and nuclear genomes of *P. falciparum*, *P. vivax*, and *P. malariae* from 16 countries spanning ~5500 years of human history.^81^ This section will explore the evolution of *Plasmodium* parasites, tracing their origins from free-living protozoa with chloroplasts to the complex parasites that depend on the apicoplast for host adaptation. It delves into the genomic characteristics of various *Plasmodium* species, highlighting differences in genome size, organization, and GC/AT content variations, as well as the extensive genome sequencing efforts listed in PlasmoDB. Comparative genomic analyses of different *Plasmodium* strains have been explored to reveal insights into genomic diversity, parasite evolution, and population genetics. Additionally, the section will review rodent malarial parasite models, such as *P. chabaudi*, *P. yoelii*, and *P. berghei*, emphasizing their conserved core genomes and the unique subtelomeric gene families that facilitate immune evasion. Finally, it highlights the advancements in single-cell biology techniques applied to *Plasmodium* research, showcasing significant findings from single-cell RNA sequencing studies that enhance researcher understanding of parasite development, transmission-blocking strategies, and host-parasite interactions.

In terms of DNA sequences*, Plasmodium* species have compact genomes of 18–30 megabases (Mb) packaged into 14 chromosomes,^82^ with multigene families commonly found near the telomeric ends of each chromosome, which are organized as heterochromatin in distinct clusters at the periphery of the nucleus.^83^ The *P. falciparum* 3D7 genome was the first malaria parasite genome to be fully sequenced and the sequencing results revealed that it has an exceptionally low GC content of under 20%.^84^ Moreover, the genomes of avian malaria parasites such as *P. relictum* and *P. gallinaceum*, which are similar to that of *P. falciparum*, have high AT contents.^85,86^ *Polychromophilus* parasites, which infect bats, have compact genomes with a small number of protein-coding and RNA genes, highlighting their unique evolutionary adaptations.^87^ By 2022, many *Plasmodium* genomes had been sequenced and deposited in the public database PlasmoDB (https://plasmodb.org/).

Comparative analyses of the genomic sequences from the field isolates of various *Plasmodium* species revealed features in genomic diversity, parasite evolution, population genetics, and drug resistance possibilities.^54^ For example, *P. falciparum* NF54, which was isolated from a patient in the Netherlands, was one of the first strains used in clinical trials for malaria vaccine study.^88,89^ Its genome size is ~23.40 Mb, with ~5273 protein-coding genes (PCGs), 229 noncoding RNA (ncRNA) genes, and 107 pseudogenes. The *P. falciparum* 3D7 strain, a parent clone of *P. falciparum* NF54, is the most widely used strain in laboratories worldwide.^53^ Its genome is ~23.33 Mb, comprising ~5318 PCGs, 244 ncRNA genes, and 158 pseudogenes. The *P. falciparum* HB3 strain is a well-characterized Honduran chloroquine-sensitive strain.^90,91^ Its genome is approximately 22.81 Mb, with ~5186 PCGs, 141 ncRNA genes, and 134 pseudogenes. The *P. falciparum* 7G8, a Brazilian isolate and genetically distinct from the West African parasite *P. falciparum* NF54,^92^ its genome is ~22.83 Mb, containing ~5183 PCGs, 161 ncRNA genes, and 161 pseudogenes. Collectively, the genomic sequences of these strains provide valuable insights into the diversity and evolution of *P. falciparum*, aiding in vaccine development and drug resistance studies.

Rodent malarial parasite species serve as valuable models for studying issues that are challenging to address with human-infecting species such as *P. falciparum* and *P. vivax*.^93^ Three commonly used laboratory species are *P. chabaudi*, *P. yoelii*, and *P. berghei*.^93^ Both human and animal malarial parasites share a highly conserved core genome.^82^ This includes essential genes for fundamental biological processes, such as replication, transcription, and basic metabolic pathways.^94,95^ In addition, both human and animal *Plasmodium* species have chromosomal subtelomeric regions that contain large gene families involved in host‒pathogen interactions and antigenic variation. These regions are prone to a high rate of recombination, aiding in gene diversity and immune evasion. For example, the *P. vivax* (human) and *P. yoelii* (rodent) genomes both feature variable gene families in subtelomeric regions. However, *P. falciparum* has a unique gene family, the *var* gene family, encoding *P. falciparum* erythrocyte membrane protein 1 (PfEMP1) proteins involved in cell adhesion and pathogenesis, which are absent in rodent and other primate malarial parasites. Similarly, rodent malarial parasites have their own unique gene families, such as the CIR/BIR/YIR families, which are absent in human malarial parasites.^94,96^

**Fig. 2:**
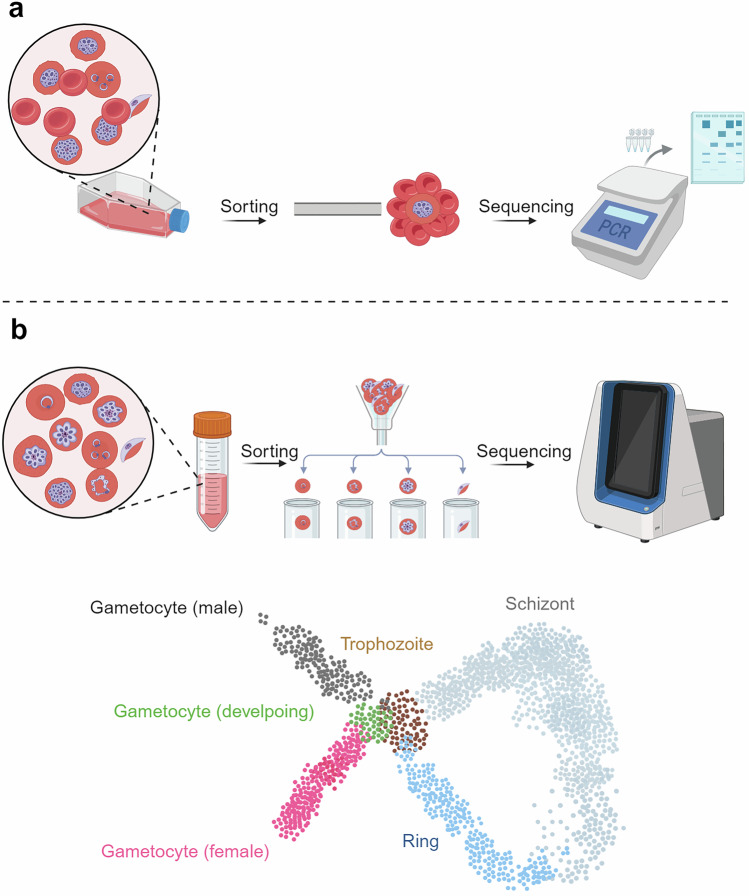
scRNA-seq analysis of *P. falciparum*. **a** A single *P. falciparum*-infected erythrocyte was manually isolated from a rosette and held by a 5-mm micropipette under a light microscope.^97^ **b** The scRNA sequences of *P. falciparum* in the asexual and sexual blood stages.^98^

Research on the *Plasmodium* genome has entered an exciting era with the development and application of single-cell biology (Table 1). In 1998, single-cell reverse transcription PCR was first applied to amplify *var* transcripts encoding PfEMP1 with degenerate primers (Fig. 2a), leading to the discovery of multiple transcription events of *var* genes in a single *P. falciparum* parasite.^97^ In 2019, Howick et al. utilized single-cell RNA sequencing (scRNA-seq) and identified 20 transcriptional modules among 5,156 key genes, revealing a high-resolution transcriptional atlas during the life cycle of *P. berghei*. The application of this atlas led to the possibility of defining all *Plasmodium* developmental stages on the basis of stage-specific transcription markers (Fig. 2b).^98^ In the ookinete stage, Witmer et al. utilized scRNA-seq to profile transcriptional variation in *P. berghei* ookinetes across different vector species and within individual midguts.^99^ The findings revealed significant clonal variation, which is crucial for understanding how ookinetes adapt to different environmental cues and how this adaptation impacts transmission-blocking strategies. Additionally, an scRNA-seq analysis revealed that hepatocyte zonation affects the development of the rodent malaria parasite *P. berghei* ANKA in the liver stage, with parasites developing more rapidly in pericentral lobule zones; moreover, this study revealed a subpopulation of periportally biased hepatocytes with abortive infections that promote immune cell recruitment.^100^

**Table 1:** Studies on *P. falciparum* at the single-cell level

Development Stage	Cell Numbers	Reference	Year
Intraerythrocytic stage	>260	^493^	2014
Intraerythrocytic stage	48	^494^	2017
Stage I and stage IV gametocytes	18,000	^495^	2017
Asexual and sexual life-cycle stages	500	^496^	2018
Intraerythrocytic stage	165	^497^	2018
Gametocyte	90	^498^	2018
Gametocyte	336	^499^	2018
Gametocyte	7472	^500^	2019
Intraerythrocytic stage	~6000	^98^	2019
Intraerythrocytic stage	46 synchronized asexual parasites	^501^	2019
Intraerythrocytic stage	315	^502^	2021
Intraerythrocytic stages	12,000	^503^	2021
Male and female gametocytes Ookinete Oocyst sporozoites Hemolymph sporozoites Salivary gland sporozoites Sporozoites released by mosquito bite Activated sporozoites	1467	^504^	2021
Blood-stage schizonts	100	^505^	2022
Zygote and ookinete stages	180	^506^	2023
Intraerythrocytic stage	10 000	^507^	2024
Intraerythrocytic asexual and sexual development stages	~45700 cells	^508^	2024

### Clinical features of malaria

Malaria presents with a wide spectrum of clinical manifestations, ranging from uncomplicated forms to severe, life-threatening complications.^101^ The clinical features of malaria are primarily influenced by the species of *Plasmodium* responsible for the infection, and timing of the diagnosis and treatment.^61^ This section will provide an in-depth analysis of the clinical manifestations primarily associated with *P. falciparum* infection, which mainly focuses on severe symptoms. It begins by outlining the range of symptoms, highlighting the complex pathologies that necessitate comprehensive management strategies. The section will then focus on CM, detailing its definition. Additionally, the section will explore pregnancy-associated malaria (PAM), emphasizing the unique mechanisms of placental sequestration and its detrimental effects on both maternal and fetal health. Pulmonary complications such as pulmonary edema and acute respiratory distress syndrome (ARDS) are also examined, with an emphasis on their pathophysiological mechanisms, differences in prevalence and presentation between adults and children, and the underlying immune responses. Finally, the multifaceted clinical features of severe malaria are summarized, integrating both direct parasite-induced effects and indirect immune-mediated processes, and potential therapeutic interventions aimed at mitigating these severe outcomes are reviewed.

In its uncomplicated form, malaria typically begins with a combination of nonspecific symptoms such as fever, chills, sweats, headaches, nausea, vomiting, muscle aches, and general malaise.^102^ These symptoms can often be mistaken for common viral infections like influenza, especially in regions where malaria is rare. However, in malaria-endemic areas, these symptoms are frequently recognized as indicative of malaria, leading to self-treatment or presumptive diagnosis. On physical examination, signs such as elevated temperature, sweating, weakness, splenomegaly, mild jaundice, hepatomegaly, and an increased respiratory rate may be observed. Diagnosis of uncomplicated malaria is confirmed through the identification of *Plasmodium* parasites in blood samples, typically using microscopy. Laboratory findings often include mild anemia, thrombocytopenia (low platelet count), elevated bilirubin, and elevated liver enzymes (aminotransferases).^102,103^ In clinics with the availability of the rapid diagnostic test, malaria can be determined.^104^

Severe malaria occurs when the infection leads to serious complications, often involving organ failure or abnormalities in the blood or metabolism.^105^ This progression typically follows delayed diagnosis or inadequate treatment. Criteria for severe malaria can vary, but in the US commonly include high parasitemia (≥5%), impaired consciousness, seizures, circulatory collapse or shock, acute respiratory distress syndrome (ARDS), acidosis, acute kidney injury, disseminated intravascular coagulation (DIC), jaundice (accompanied by at least one other sign), severe anemia (hemoglobin <7 g/dL).^102^

In *P. vivax* and *P. ovale* infections, patients who have recovered from an initial episode may experience relapses months or even years later.^106^ These relapses are caused by the dormant liver-stage parasites, known as hypnozoites, which can reactivate and initiate new cycles of infection.^106^

Malaria can lead to a variety of other complications. Neurological deficits,^107,108^ such as ataxia, palsies, speech difficulties, hearing loss, cognitive impairments, and blindness, may persist after cerebral malaria, particularly in children. Recurrent infections with *P. falciparum* may result in severe anemia,^109^ especially in young children in tropical regions. Pregnancy-related malaria,^110^ particularly caused by *P. falciparum*, can lead to severe disease in the mother, premature delivery, or low birth weight infants. Rare complications include splenic rupture in *P. vivax* infections and nephrotic syndrome due to chronic *P. malariae* infections.^111^

Periodic fever, a hallmark of *falciparum* and *vivax* malaria, is linked to erythrocyte rupture accompanied by the release of hemozoin after each erythrocytic cycle and the host’s inflammatory response.^112^ The periodicity of fever is determined by the replication cycle of the parasite within RBC.^112–114^ In *P. falciparum*^115^ and *P. vivax*,^116^ the fever cycle is typically 48 h (known as the tertian cycle), while in *P. malariae*, the fever cycle extends to 72 h (fever regularly occurs again on the fourth day in many patients, quartan cycle).^117^ The synchronized rupture of RBCs at these intervals leads to the periodic nature of fever, which typically follows a “chill-fever-sweat” pattern (An attack usually starts with shivering and chills, followed by a high fever, sweating, and a return to normal temperature). In addition to the characteristic periodic fever, anemia in malaria is primarily due to the destruction of both iRBCs and uninfected RBCs.^118^ Thrombocytopenia, often observed in individuals with malaria, results from both the direct destruction of platelets and splenic sequestration.^119^ Renal impairment, including acute kidney injury (AKI), can occur due to systemic inflammation and direct effects of the parasitic infection on the kidneys.^120^ These complex pathologies underscore the need for comprehensive management strategies in severe malaria patients to address the multifaceted pathological effects.

**Fig. 3:**
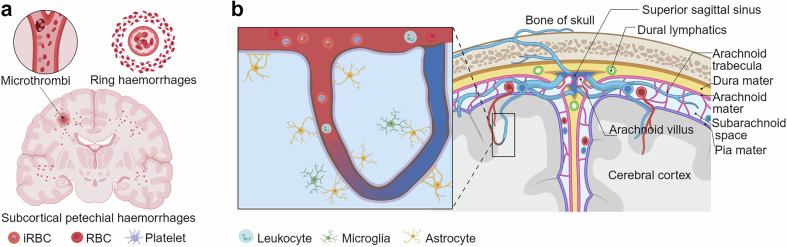
Pathophysiology of CM. **a** Subcortical petechial hemorrhages and microthrombi formation in the brain that occur during CM often result in ring hemorrhages and microvascular damage. **b** The interaction between iRBCs and endothelial cells in the cerebral vasculature. This figure is created with BioRender.com

CM is the most severe form of *P. falciparum* infection and mostly occurs in children under 5 years of age in malaria-endemic areas.^108^ It is defined as a microscopically confirmed *P. falciparum* infection and a Blantyre coma score ≤2, with no other known cause of coma.^121^ The sequestration of iRBCs in brain capillaries and postcapillary venules is the cause of cerebral hypoxia and coma.^122^ Additionally, the defining factor of CM is the formation of rosettes by *P. falciparum* erythrocyte membrane protein 1 (PfEMP1) binding to uninfected erythrocytes.^123^ A study showed that *P. falciparum* isolates from children with CM consistently form erythrocyte rosettes and lack anti-rosette antibodies, whereas isolates from children with mild malaria exhibit reduced or no rosettes and are disrupted by anti-rosette antibodies, thereby supporting the role of erythrocyte rosetting in the pathogenesis of CM and the protective effect of anti-rosette antibodies.^124^ Another study showed that specific PfEMP1-duffy binding-like domain 1 (DBL1α) motifs are correlated with rosetting and severe malaria, suggesting that *P. falciparum* strains with particular PfEMP1 sequences cause severe malaria.^125^ The pathophysiological processes underlying CM involve substantial microvascular changes, including ring hemorrhages, microthrombi, and fibrin deposits, predominantly in the white matter and border zones between the major cerebral arteries (Fig. 3a, b).^126^ These structural changes have been found to be caused by coagulation defects in both murine experimental cerebral malaria (ECM) and human CM.^127^ Brain swelling, associated with cerebral vasculature sequestration, is a leading cause of death in CM.^128^ Among 348 children admitted with CM (as defined by the WHO), 168 met the inclusion criteria and were included in a correlation analysis. Of these, 25 children (15%) died, 21 of whom (84%) had severe brain swelling on magnetic resonance imaging (MRI) at admission, whereas only 27% (39 of 143) of the survivors had similar swelling.^129^ Serial MRI scans of survivors initially presenting with brain swelling revealed a decrease in brain volume postinfection.^129^ The mechanisms proposed for this swelling include cytotoxic edema caused by cellular injury and swelling and vasogenic edema resulting from disruption of the blood‒brain barrier (BBB) and leakage of plasma into the brain.^130,131^ High-resolution MRI studies suggest that vasogenic edema is a predominant feature of CM that can be rapidly alleviated with treatment.^132,133^ These findings align with the characteristics of posterior reversible encephalopathy syndrome,^134^ highlighting potential endothelial dysfunction and impaired autoregulation in CM.

Pregnancy-associated malaria (PAM), also known as placental malaria, is caused by *P. falciparum* parasites that express a specific PfEMP1 variant (VAR2CSA) only in pregnant women, enabling placental sequestration by the parasites through binding to the placental ligand chondroitin sulfate A (CSA).^135–137^ This sequestration leads to damage to the placenta, as well as adverse effects on both the fetus and the mother. PAM is a significant public health concern, particularly in malaria-endemic regions.^138^ Adult residents of malaria-endemic regions typically develop immunity to malaria through repeated exposures to malaria parasites. However, malaria poses a unique and heightened risk to pregnant women, especially to those experiencing the first pregnency.^139^ The majority of malaria infections during pregnancy remain asymptomatic or paucisymptomatic yet are a major cause of severe maternal anemia and preventable adverse outcomes for both mothers and infants, especially in the first and second pregnancies.^139^ Despite the implementation of preventive measures such as intermittent preventive treatment with sulfadoxine-pyrimethamine (SP), many pregnant women are unaware of these preventative treatments, and patient adherence to these interventions can be poor. Studies have shown that even with high attendance at antenatal care clinics, the prevalence of asymptomatic *P. falciparum* infections among pregnant women remains high, contributing to maternal anemia and low birth weight in newborns.^140,141^ The structural basis for the interaction between Var2CSA and CSA has been elucidated through advanced techniques such as cryo-electron microscopy, revealing that Var2CSA has a unique architecture that facilitates its binding to CSA.^142,143^ Specifically, Var2CSA interacts with CSA by binding within two distinct channels that traverse the core domain. Importantly, binding to CSA does not induce significant conformational changes in the Var2CSA protein, maintaining its structural integrity during the adhesion process. Furthermore, the phosphorylation of Var2CSA has been identified as a critical factor that enhances its adhesive properties to CSA, indicating that posttranslational modifications can influence the virulence of the parasite.^144^

**Fig. 4:**
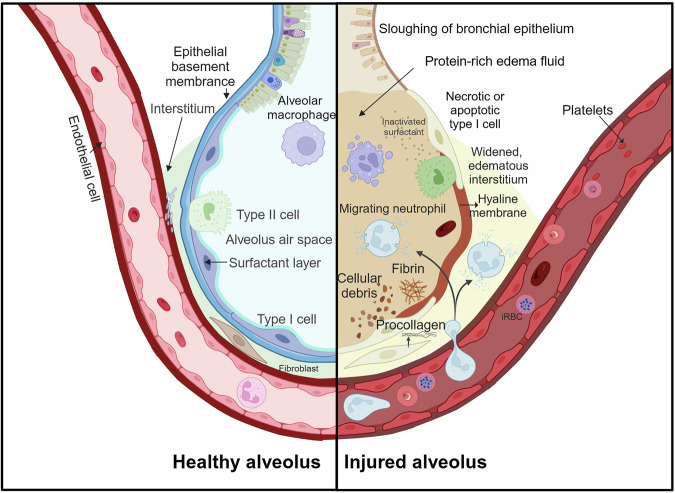
Comparison of healthy and injured alveoli in malaria-induced acute lung injury. The healthy alveoli show intact epithelial and endothelial barriers, clear alveolar air spaces, and functional type I and II cells. In contrast, the injured alveoli display sloughing of the bronchial epithelium, protein-rich edema fluid, and necrotic type I cells. This figure was created with BioRender.com

Pulmonary complications in *P. falciparum* malaria patients primarily manifest as pulmonary edema and acute respiratory distress syndrome (ARDS).^145^ Pneumonia, often caused by bacterial or viral infections, is also common in malaria patients. However, few clinical or histopathological studies have focused specifically on lung complications. ARDS is characterized by diffuse lung inflammation, alveolar damage (Fig. 4), as evidenced by poor oxygenation and radiological images of diffuse lung involvement.^146^ It is well recognized in adults with severe malaria, although its incidence varies widely.^147^ ARDS often occurs late in the disease course, even after antimalarial treatment has begun.^148^ Ultrastructural analysis of the lungs of Asian adults with severe malaria and ARDS revealed classic features, such as hyaline membranes and neutrophil and monocyte infiltration, accompanied by significant fibrin formation (Fig. 4).^149^ Furthermore, postmortem studies in Vietnamese adults with fatal severe malaria revealed marked loss of EPCR and thrombomodulin in the lungs, similar to findings in children with CM, indicating a shared pathophysiological mechanism^150^ Pulmonary edema is typically linked to fluid overload from excessive intravenous fluids, heart failure, or renal failure and may be exacerbated by increased vascular permeability (Fig. 4).^151^ ARDS and pulmonary edema occur less frequently in children than in adults.^152^ Data from the Fluid Expansion as Supportive Therapy (FEAST) study indicate that fluid administration in children can increase mortality, with post hoc analysis suggesting respiratory deterioration as a key mechanism.^153^ This implies that children with CM may have an increased, although lower than that of adults, risk of capillary leakage in the lungs. Other studies supported this result. In children, respiratory distress is often associated with acidosis rather than hypoxia, but ARDS^154^ and pulmonary edema^155^ are rare, indicating compensatory hyperventilation rather than lung pathology. The absence of hyaline membranes or alveolar damage in pediatric autopsy studies suggests that lung pathology in children may be subclinical and detectable only postmortem, indicating greater lung vulnerability in adults than in children.^156^

Overall, the pathophysiology of malaria is multifaceted and involves both direct and indirect mechanisms. The direct effects of *P. falciparum* include the sequestration of iRBCs in the pulmonary microvasculature, leading to microvascular obstruction, endothelial activation, and subsequent inflammatory responses.^157,158^ This sequestration is also mediated by the interaction of parasite-derived proteins such as PfEMP1 with endothelial receptors such as ICAM-1 and EPCR, resulting in endothelial cell activation and disruption of the endothelial barrier.^125^ Importantly, depolymerized glycosaminoglycans (dGAGs) lacking anticoagulant activity have been identified as promising candidates for adjunct therapy in severe malaria.^159^ These dGAGs effectively disrupt rosette formation, inhibit merozoite invasion and endothelial binding, and reduce sequestration of *P. falciparum*-infected erythrocytes in the nonhuman primate *Macaca fascicularis*.^159^ The indirect effects involve systemic inflammatory responses, where cytokines such as TNF-α and IFN-γ may play critical roles in exacerbating endothelial permeability and promoting leukocyte recruitment to the lungs.^160^ Neutrophils, monocytes, and other immune cells are recruited to the lungs, where they release inflammatory mediators and proteolytic enzymes that contribute to tissue damage. The formation of neutrophil extracellular traps (NETs) and the release of reactive oxygen species further damage the alveolar‒capillary barrier, promoting edema and impairing gas exchange.^161^ Thus, understanding these mechanisms is crucial for developing targeted interventions to mitigate lung damage and improve outcomes in severe malaria patients.

### Crosstalks between *Plasmodium* and host red blood cells

#### Regulatory mechanisms governing Plasmodium development

**Fig. 5:**
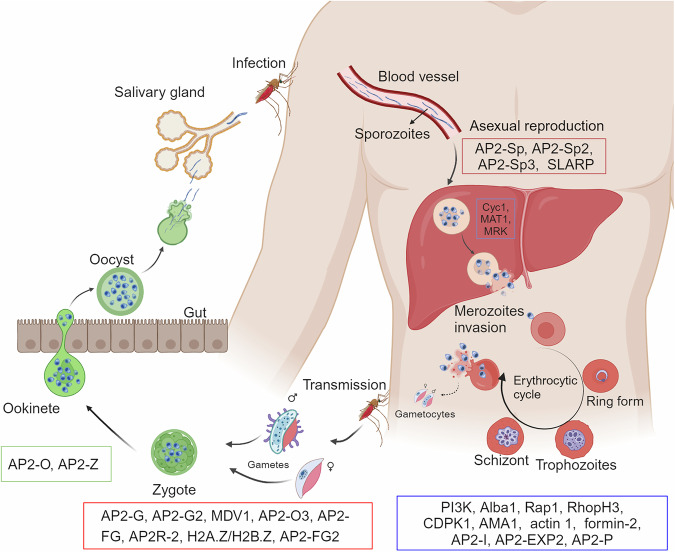
The life cycle of *P. falciparum* and the known regulatory proteins associated with parasite development and differentiation. The *Plasmodium* parasite has a complex life cycle in both human and mosquito hosts. This figure was created with BioRender.com

The complex life cycle of *Plasmodium* species involves repeated transmission between mosquitoes and vertebrate hosts (Fig. 5).^162^ This section outlines the complex lifecycle of the parasites, from gametocyte formation in the human host to sporozoite development in mosquitoes and subsequent infection of hepatocytes in mammalian hosts. It emphasizes the critical roles of various transcription factors in regulating stages of gametocytogenesis, ookinete formation, and sporozoite development. Additionally, this section explores host invasion mechanisms, detailing the multistep process of erythrocyte invasion by merozoites, the involvement of merozoite surface proteins, erythrocyte binding antigens, and the formation of tight junctions mediated by the apical membrane antigen 1 (AMA1) -RON complex.

Gametocytes are the first life forms of the sexual phase in the *Plasmodium* parasite life cycle,^163^ which are critical for parasite dissemination (Fig. 5). The transcription factor (TF), *P. falciparum* apetala2 gametocyte (PfAP2-G), is involved in the regulation of gametocytogenesis and sexual commitment in *P. falciparum*,^164^ which orchestrates the gametocyte stage.^165^ Moreover, conditional expression of PfAP2-G enables the characterization of the early sexual stages of the parasite, including sexually committed schizonts and sexual rings, and reveals key changes, such as the downregulation of genes involved in solute transport upon sexual commitment.^166^ Additionally, PfAP2-G2 significantly modulates the production and maturation of gametocytes by regulating the expression of *P. falciparum* male development gene 1 (MDV-1).^167^ Furthermore, *P. falciparum* apetala2 ookinetes 3 (PfAP2-O3) acts as a repressor in female gametocytes, ensuring sex-specific gene expression.^168^ AP2-O3-deficient parasites produce apparently normal female gametocytes, but these gametocytes fail to differentiate, leading to developmental arrest after fertilization.^168^ In *P. berghei*, a rodent malarial parasite, apetala2-female specific (AP2-FG) has been reported as a TF for female-specific gene regulation, emphasizing its role in the development of female gametocytes.^169^ In addition to the AP2 transcription factor, histone variants and histone modifications also play roles in sex specification in parasites.^170,171^ Specifically, in female gametocytes, the *P. falciparum* histone variants PfH2A.Z/H2B. Z are highly enriched in histone H3 lysine 9 trimethylation (H3K9me3)-associated heterochromatin.^172^

These mature gametocytes are taken up by mosquitoes during a blood meal, leading to the mosquito stage of the parasite’s life cycle. Gametogony and sporogeny are the most important stages of *Plasmodium* development in mosquitoes (Fig. 5). In this stage, *Pb*AP2-FG2 and AP2R-2 form a transcriptional repressor complex essential for female gametocyte development, with disruptions in the formation of this complex leading to developmental arrest during ookinete formation.^173^ AP2-O is a TF that is expressed in several ookinete stages, from retort ookinetes to mature ookinetes, and activates the majority of known ookinete genes.^174,175^ Thus, disruption of AP2-O results in the impaired development of ookinetes. Moreover, *P. falciparum* apetala2 zygote (AP2-Z) is a novel TF crucial for ookinete development, with AP2-Z-mediated transcription in zygotes essential for ookinete formation; additionally, the targets of AP2-Z overlap with those of AP2-O.^176^

The development of ookinetes and oocysts in mosquitoes leads to the production of sporozoites, which are the parasite form that infects mammalian hosts.^177^ Four TFs, including AP2-sporozoite (Sp),^178,179^ AP2-Sp2,^178^ and AP2-Sp3,^178^ which are asparagine-rich proteins (SLARPs),^180^ have been reported to play important roles in gene regulation during this stage. AP2-Sp maintains its own expression via a transcriptional autoactivation mechanism (positive-feedback loop) and activates other transcription factors, including AP2-Sp2, AP2-Sp3, and SLARP, at this stage.^181^

Upon entering the mammalian host, sporozoites infect hepatocytes, eventually leading to the release of thousands of merozoites that invade RBCs. The asexual replication cycle of *P. falciparum* in erythrocytes is characterized by sequential transformations from rings (0–10 h) to trophozoites (10–40 h) and schizonts (40–48 h)^182^ (Fig. 5). As the parasite develops inside RBCs, it alters host erythrocyte biomechanical properties, notably reducing iRBC deformability.^183^ Our previous findings indicate that *P. falciparum* remodels the erythrocyte cytoskeleton through *P. falciparum* phosphoinositide 3-kinase (PfPI3K)-regulated ubiquitination and degradation of α-spectrin, a process that facilitates egress of mature parasites from iRBCs.^184^ In addition, many proteins and posttranslational modifications have been shown to be involved in regulating the asexual replication cycle.^185^ For example, *Pf* DNA/RNA-binding protein (ALBA1) can bind to four mRNA transcripts encoding erythrocyte invasion-associated proteins, including rhoptry-associated protein 1 (Rap1), rhoptry neck protein 3 (RhopH3), calcium-dependent protein kinase 1 (CDPK1), and apical membrane antigen 1 (AMA1), which are important regulators of the translational timing and asexual proliferation of *P. falciparum*.^186^ Pfactin1^187^ and Pfformin-2^188^ are actin-related proteins that are essential for proper and efficient segmentation in iRBCs and involve the structural organization necessary for cell division. PfCyc1, a cyclin H homolog, along with its potential partners PfMAT1 and MO15-related protein kinase PfMRK, are critical for merozoite formation and development.^189^ Parasites lacking PfCyc1 can still form nuclei and apical organelles but fail to produce merozoites.^189,190^ In addition, the PfAP2-invasion (PfAP2-I) factor, which belongs to the Apicomplexan AP2 family, is responsible for regulating the expression of genes involved in RBC invasion.^191^ Furthermore, PfAP2-EXP2 regulates genes associated with parasite virulence and host‒parasite interactions.^192^ A recent study revealed that the expression of the essential TF PfAP2- pathogenesis (P), which critically regulates the parasite transition from trophozoites to schizonts, peaks during two phases of the blood-stage development of *P. falciparum*. The underlying mechanism involves PfAP2-P binding to the promoters of genes controlling trophozoite development and host cell remodeling.^193^ Additionally, the inhibition of N-myristoyl transferase (NMT) in *P. falciparum* disrupts parasite development and growth at multiple stages, including schizogony, rhoptry formation, merozoite egress, and erythrocyte invasion, highlighting NMT as a critical drug target due to the pleiotropic effects of its inhibition.^194^ Moreover, the critical role of IMC1g proteins in the *Plasmodium* parasite life cycle, specifically PbIMC1g in *P. berghei*^195^ and its functional counterpart, PfIMC1g,^196^ in *P. falciparum* has been recognized. PbIMC1g is involved in asexual replication, gametogenesis, ookinete motility, and mosquito midgut invasion, confirming its role in maintaining structural integrity and facilitating parasite motility during invasion. In *P. falciparum*, PfIMC1g is essential for the asexual replication stage, as its deficiency leads to parasite death shortly after red blood cell invasion. The evolutionary conservation of IMC1g proteins across *Plasmodium* species also indicates that these proteins could be key targets for therapeutic interventions. Overall, understanding these regulatory mechanisms across life cycle stages is crucial for developing effective malaria control and treatment strategies.

#### Host cell invasion mechanisms of malarial parasites

Once released from schizonts, merozoites may take several seconds or minutes before establishing contact with the surface of an RBC and commencing invasion. *Plasmodium* merozoites, previously thought not to exhibit gliding motility, can indeed undergo this movement in vitro, a crucial step for successful invasion.^197^ After primary attachment of the merozoite to the RBC surface, invasion occurs within ~30 s.^198,199^ The invasion of RBCs by *P. falciparum* merozoites is a complex, multistep process involving numerous parasite proteins and host RBC surface receptors. This invasion process can be achieved through two distinct pathways: (1) the sialic acid (SA)-dependent pathway, where proteins such as erythrocyte binding antigen 175 (EBA-175), erythrocyte binding ligand 1 (EBL-1) bind to glycophorin A and EBA-140 bind to glycophorin C^200,201^ on the RBC surface; and (2) the SA-independent pathway, where proteins such as *P. falciparum* reticulocyte binding protein homolog 5 (RH5) and PfRh4 interact with receptors such as complement receptor 1 (CR1), basigin (also known as CD147), and glycophorin A (GYPA), enabling invasion without requiring SA.

**Fig. 6:**
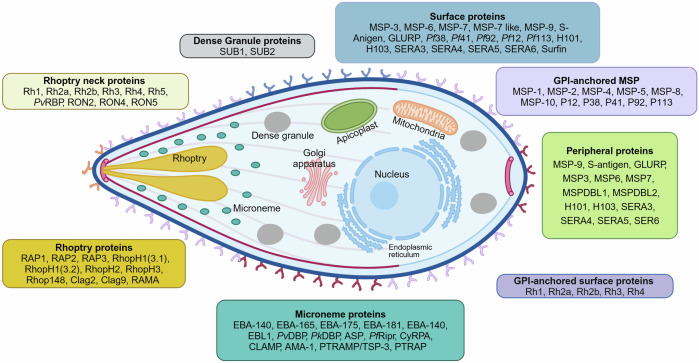
Merozoite proteins involved in erythrocyte invasion of *P. falciparum* parasites. The various protein groups associated with different organelles in the *Plasmodium* parasite were shown. It categorizes key proteins into distinct groups, including rhoptry proteins, dense granule proteins, surface proteins, GPI-anchored surface proteins, peripheral proteins, and microneme proteins. Each protein group is color-coded for clarity and shown in association with the relevant organelle or cellular structure. This figure was created with BioRender.com

**Fig. 7:**
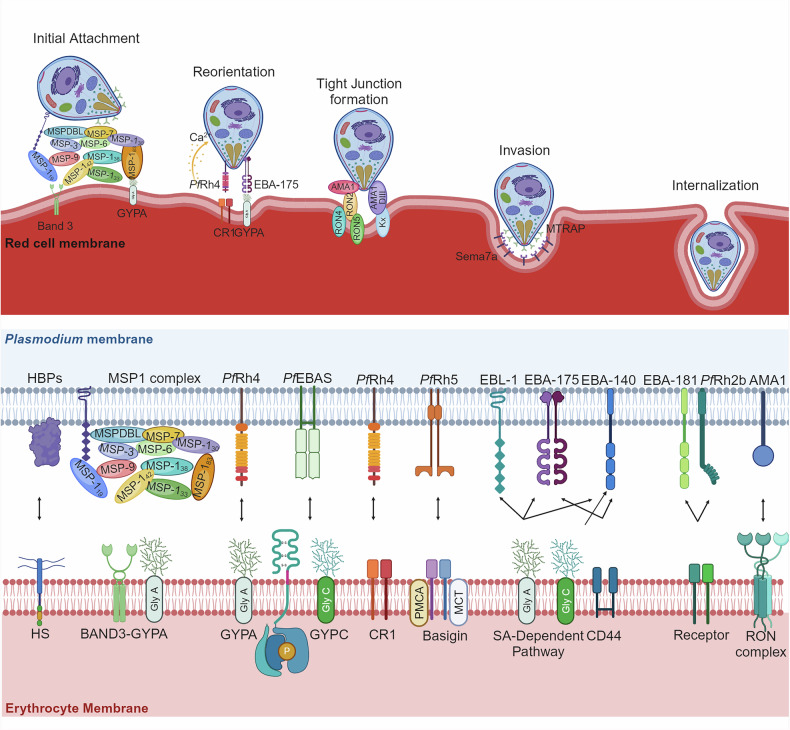
Mechanisms of *Plasmodium* invasion in erythrocytes. In the upper panel, the sequence of invasion begins with the attachment of the merozoite to the erythrocyte surface, followed by the discharge of the microneme and rhoptry contents, leading to apical reorientation, tight junction formation and erythrocyte membrane invagination, and the eventual entry of the merozoite into the erythrocyte. The lower panel highlights the key protein interactions during this process, revealing two distinct invasion pathways. HBP heparin-binding proteins, HS heparan sulfate, SA sialic acid, SA sialic acid, GYPA glycophorin A, GYPA glycophorin C, CR1 complement receptor 1, MSPs merozoite surface proteins, EBA erythrocyte binding antigen, EBL erythrocyte binding ligand, Rh reticulocyte binding protein homolog, AMA1 apical membrane antigen 1. This figure was created with BioRender.com

The initial attachment to the RBC surface is mediated by merozoite surface proteins (MSPs), such as MSP1,^202^ MSP2, MSP6,^203^ and MSP9 (orthologous to p101/ABRA of *P. falciparum*),^204^ along with other glycosyl phosphatidylinositol (GPI)-anchored MSPs (Figs. 6 and 7). MSP1 is the most abundant merozoite surface protein anchored on the merozoite surface (Fig. 6).^205^ It essentially mediates erythrocyte invasion via interactions with host glycophorin A^206^ and heparin-like molecules.^207^ Recently, a study revealed that t a highly basic region within the central cavity of MSP1 may promote weak adhesion to erythrocytes via long-range electrostatic interactions, specifically targeting negatively charged heparin-like polysaccharides abundant on the erythrocyte surface.^208^ The posttranslational modification and processing of MSP1 by the parasite protease SUB1, which is released from dense granules, are necessary steps in merozoite maturation.^185,209^ Initially, expressed as a high-molecular-weight protein (∼200 kDa), MSP1 undergoes primary proteolytic processing, resulting in four fragments (83 kDa, 30 kDa, 38 kDa, and a C-terminal 42 kDa segment) that form a noncovalent complex on the merozoite surface.^210^ This complex mediates the initial attachment of the merozoite to RBCs through interactions with heparin-like proteoglycans or Band 3 proteins, facilitating successful invasion.^211^ Following egress from the host cell, MSP1 is further cleaved at a juxtamembrane site by *P. falciparum* subtilisin-like protease 2 (*Pf* SUB2), leading to shedding of the majority of the MSP1 complex, with only a 19 kDa C-terminal region (MSP1_19_) attached to the merozoite surface.^212^ The precise timing and spatial regulation of these processing events are governed by the discharge of subtilisin-like protease 1 (SUB1),^209^ which is activated by plasmepsin X through the cleavage of SUB1 inhibitory segments.^213^ Recently, a study shown that membrane-bound protease SUB2 is essential for the shedding of surface proteins during *Plasmodium* merozoite invasion into RBCs. Genetic depletion of SUB2 disrupts this process, leading to abortive invasion or developmental arrest.^214^ Heparin and heparan sulfate (HS), members of the glycosaminoglycan (GAG) family and consist of repeating disaccharide units of β-glucuronic acid (GlcA) and α-N-acetylglucosamine (GlcNAc),^207^ can interact with MSP1_33_. They inhibit *P. falciparum* growth and merozoite invasion by interacting with a variety of merozoite-derived proteins, and the use of structurally defined modified K5 polysaccharides enables the investigation of the specific structural requirements of antimalarial drugs to exert a robust therapeutic effect.^207,215^ Furthermore, heparin-like GAGs, such as heparan sulfate, are receptors in parasite rosettes,^216^ and rosettes may assist newly egressed merozoites in invading surrounding RBCs.^217^ Our laboratory used two-dimensional liquid chromatography‒mass spectrometry to identify 811 schizont-derived proteins that bind strongly to heparin, and those exhibited most affinity to heparin are merozoite-derived proteins.^215^ Heparin-like GAGs are likely common receptors for *Plasmodium* parasites, as numerous merozoite proteins from *P. berghei* have also been found to interact with these GAGs^218^ Therefore, heparin can be developed as an antimalarial drug or as a carrier for the targeted delivery of other antimalarial agents.^219^ Additionally, although its receptor‒ligand interaction^199^ remains further exploration, another MSP member, MSP2, is essential for invasion and is characterized by its dimorphic nature and propensity to form fibrils.^220^

Following initial attachment, the merozoite reorients itself so that its apical end faces the erythrocyte membrane. This reorientation is crucial for successful invasion and is mediated by microneme proteins such as erythrocyte binding antigen 175 (EBA175).^221,222^ Moreover, *Plasmodium* erythrocyte binding antigen (EBA) families are generally thought to play a role in the later stages of invasion, but some members may be presented on the merozoite surface in a regulated manner after the initial merozoite–erythrocyte contact has occurred (Figs. 6 and 7).^223^ Low potassium ion concentrations trigger an increase in cytosolic calcium levels in *P. falciparum* merozoites, leading to the sequential secretion of EBA-175.^224^ The crystal structure of the erythrocyte-binding domain of EBA-175 revealed its dimeric organization with critical glycan binding sites, highlighting the significant role of the F2 domain in cytoadherence (Fig. 7).^225^ Furthermore, EBA-175 protein shed from *P. falciparum* promoted the clustering of RBCs through a glycophorin A-dependent mechanism (Fig. 7), facilitating parasite growth by providing daughter merozoites with access to uninfected RBCs and protecting the invasion machinery from immune recognition.^226^ Recent studies have shown that *P. falciparum* utilizes CD44 as a coreceptor during erythrocyte invasion, with EBA-175 and EBA-140 binding to CD44 and inducing CD44-dependent phosphorylation of host cytoskeletal proteins, which enhances parasite entry by altering erythrocyte deformability.^227^ However, different malaria parasite clones utilize distinct invasion pathways, including the utilization of a glycophorin B-dependent, sialic acid-dependent pathway that operates independently by EBA-175.^228^ *P. falciparum* also employs multiple polymorphic ligands, including JESEBL/EBA-181 and EBA-140, to recognize various receptors on the erythrocyte surface, demonstrating a high level of invasion adaptability that contrasts with the single-pathway invasion strategy of *P. vivax* and contributes to its success in endemic regions.^229,230^ EBA-140 specifically binds to glycophorin C through its binding region (Region II) (Fig. 7), highlighting the role of specific glycophorin C regions and glycans in this interaction.^230^ The inactivation (pseudogenization) of the EBA165 gene in *P. falciparum*, which originally encoded an erythrocyte invasion protein specific to ape erythrocytes, was a key evolutionary step that allowed the parasite to adapt to human hosts by avoiding incompatibility with human erythrocytes.^231^ Moreover, PfRH2a/2b proteins are critical for *P. falciparum* erythrocyte invasion through distinct sialic acid-dependent and independent pathways, with their conserved N-terminal receptor-binding domain being a promising target for malaria vaccine development.^232^ Other studies investigated the prevalence of a 0.58 kbp deletion in the *PfRh2b* gene in *P. falciparum* populations, which is linked to immune evasion. The deletion is widespread across various transmission areas in Ghana and globally, with a significant frequency in hyper-endemic regions, and its presence correlates with lower immune recognition, as shown by antibody levels similar to those against PfRh5.^233^

Following reorientation, tight junctions are formed through high-affinity interactions between apical membrane antigen 1 (AMA1) and the rhoptry neck protein complex, thereby linking the merozoite surface with the erythrocyte membrane (Fig. 7).^234^ The AMA1-RON complex is also crucial for the invasion of *Plasmodium* sporozoites into mosquito salivary glands and mammalian host hepatocytes, with its absence leading to impaired colonization and altered entry junction morphology.^235^ Research on the related parasite *T. gondii* suggests that RON2 integrates into the host membrane, where it acts as the receptor for AMA1, a mechanism used by all apicomplexan parasites to facilitate invasion through their own ligand‒receptor interactions.^236^ Interestingly, blocking the AMA1-RON2 interaction inhibited tight junction formation but still resulted in erythrocyte echinocytosis, suggesting that tight junction formation follows the engagement of reticulocyte binding protein homolog 5 and the signaling events triggered by rhoptry release.^237^

With tight junctions established, the merozoite invades the erythrocyte through a process involving rhoptry proteins and the formation of a parasitophorous vacuole (PV). Rhoptry neck proteins such as (reticulocyte binding protein) Rh1, Rh2b, and rhoptry neck protein 2 (RON2), as well as rhoptry associated protein 1 (RAP1) and RhopH3, contribute to the establishment of the parasitophorous vacuole and subsequent modification of the host cell for parasite development.^238^

### Pathogenetic mechanisms

The pathogenesis of malaria, particularly *P. falciparum* infection, involves intricate molecular mechanisms that lead to severe clinical outcomes. This section highlights the role of cytokines like TNF-α and IFN-γ in activating endothelial cells, leading to the sequestration of infected red blood cells (iRBCs) via the PfEMP1 protein, a key factor in CM. It concludes how the PfEMP1 family enables the parasite to evade the immune system through antigenic variation, allowing it to adhere to host receptors such as CD36, ICAM-1, PECAM-1, and EPCR, which are associated with severe malaria. The section also covers the immune response, noting the roles of innate immune cells like macrophages and dendritic cells in producing inflammatory cytokines, and adaptive immune components such as CD4⁺ T cells, CD8⁺ T cells, and antibodies. It further describes the challenges of antigenic variation and the difficulty in achieving long-term immunity and vaccine development.

**Fig. 8:**
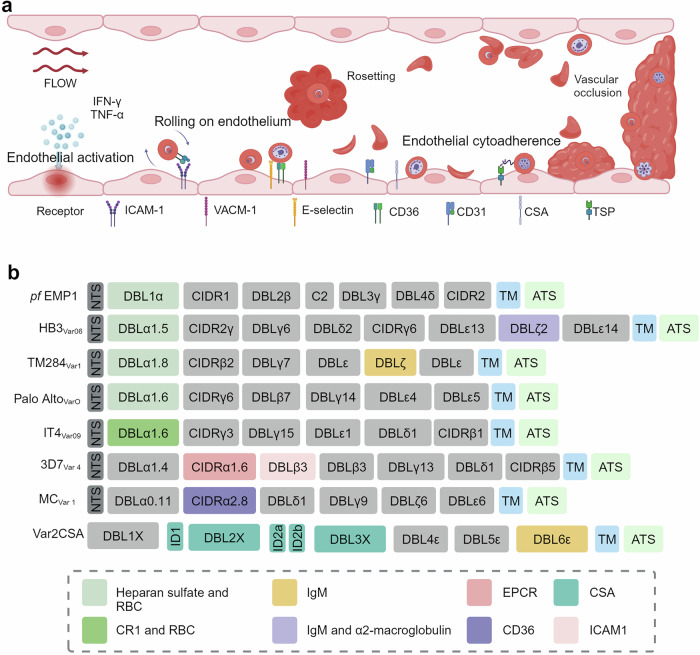
Endothelial cell activation and *P. falciparum* erythrocyte membrane protein 1 (PfEMP1)-mediated sequestration of iRBCs. **a** Mechanistic overview of the process by which activated endothelial cells express receptors that mediate the rolling and eventual sequestration of iRBCs. **b** PfEMP1 variants interact with distinct endothelial cell receptors. The PfEMP1 variants have been reviewed by Mats Wahlgren.^509^

The primary processes of sequestration of *P. falciparum*-infected erythrocytes in the microvasculature involve the activation of endothelial cells mediated by various cytokines and the adherence of iRBCs to multiple host receptors via PfEMP1 (Fig. 8a). Tumor necrosis factor-alpha (TNF-α)^239–241^ and interferon-gamma (IFN-γ)^242–244^ play critical roles in endothelial activation by upregulating the expression of endothelial adhesion molecules, thereby facilitating the sequestration of iRBCs (Fig. 8a). Additionally, the release of cytokines by immune effector cells contributes to the procoagulant state of the brain observed in patients with CM.^245^ A recent study revealed that CD8^+^ T cells adhere to the endothelium and that their interaction with perivascular macrophages leads to the release of cytotoxic cytokines, further damaging the BBB and contributing to brain edema.^246^ Mechanistically, the NH2-terminal head structure containing the duffy binding-like domain 1 (DBL1α), cysteine-rich interdomain region (CIDR1α) and DBL2δ of PfEMP1 mediates iRBC adherence to multiple host receptors (Fig. 8a),^247^ including cluster of differentiation 36 (CD36), intercellular adhesion molecule 1 (ICAM-1), platelet endothelial cell adhesion molecule-1 (PECAM-1), and endothelial cell protein c receptor (EPCR), which are closely associated with the occurrence of CM.^3,245,248^ This is discussed in more detail in the following paragraph. The binding of iRBCs to these receptors triggers a cascade of inflammatory responses and endothelial activation, contributing to the pathophysiological changes observed in CM.^249,250^

The sequestration of *P. falciparum*-infected iRBCs in the microvasculature has been recognized as the main cause of organ failure in patients with severe malaria.^251^ As previously discussed, PfEMP1, encoded by the ~60 *var* gene family, is the principal molecule implicated in CM and has been extensively characterized in the context of malaria pathogenesis.^252^ After synthesis, PfEMP1 is exported to the surface of infected red blood cells, where it forms knob structures that facilitate iRBC attachment.^253^ Although multiple distinct *var* gene transcripts can be detected simultaneously in bulk cultures and in individual infected erythrocytes, only one *var* transcript is virtually expressed and translocated on the surface of an iRBC. Moreover, frequent expression switching of these transcripts, which is mutually exclusive,^254^ results in almost unlimited strategies for the parasite to escape immune recognition and clearance.^255^

On the basis of sequence homology in the upstream regions, the *var* genes can be categorized into five subgroups: UpsA, UpsB, UpsC, UpsD, and UpsE.^256^ These subgroups are distributed across different locations on *P. falciparum* chromosomes. The UpsA subgroup *var* genes are located in the subtelomeric regions of the chromosomes; UpsB subgroup genes can be found in either telomeric or central regions; and UpsC subgroup genes are located primarily in the central regions of the chromosomes.^257^ Severe malaria is frequently associated with the expression of A or B subgroup *var* genes,^258^ whereas mild or asymptomatic malaria is linked to the expression of C subgroup *var* genes.^259^ In the protein structure (Fig. 8b), PfEMP1 contains multiple Duffy-binding-like (DBL) domains and a cysteine-rich interdomain region (CIDR) in its extracellular sequence, along with a shorter acidic terminal sequence in its cytoplasmic tail. CD36 is a receptor for most N-terminal DBL–CIDR domain cassettes across various PfEMP1 variants, a common feature of the majority of PfEMP1 variants (types B and C).^260,261^ Another receptor common to the PfEMP1 A and B types is ICAM-1.^250,262^ Antibodies against the PfEMP1 NTS-DBL1α domain can inhibit rosette formation and cytoadherence of iRBCs.^263^ Moreover, antibodies against the PfEMP1 head structure DBL-CIDR domain are more indicative of malaria exposure than are those against the DBL-α tag,^264^ offering insights into exposure and immunity dynamics. Moreover, the binding of PfEMP1 to nonimmune IgM and α2-macroglobulin (α2M) on the surface of immune cells hinders immune recognition of iRBCs, manipulates host responses, and aids in immune evasion.^265^ Additionally, experimental vaccines using virus-like particles (VLPs) conjugated to PfEMP1 domains have shown promise in inducing inhibitory antibodies, offering a potential pathway for developing effective malaria vaccines.^266^ Recently, the breadth of antibody responses to *P. falciparum* variant surface antigens on iRBCs, not to specific PfEMP1 antigens, has also been implicated as a predictive factor for protection against malaria in controlled human malaria infection.^267^

### Host immune responses to malaria

**Fig. 9:**
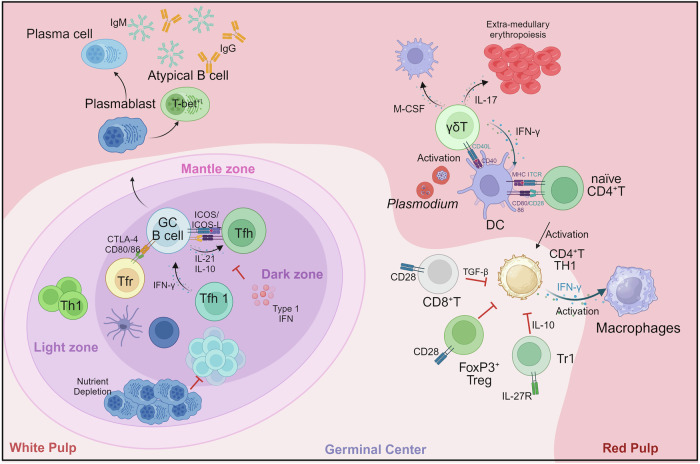
The immune responses during *Plasmodium* infection. The immune responses within the spleen during *Plasmodium* infection are shown. This figure was created with BioRender.com

The pathogenesis of malaria is closely linked to the host immune response, which affects the severity and outcome of the infection. The immune response to malaria is complex and involves both innate and adaptive responses. Initially, the innate immune system mounts a nonspecific defense,^268^ primarily through macrophages and dendritic cells, which identify infected cells and produce inflammatory cytokines such as TNF-α and IL-6.^269^ These cytokines are critical for early parasite control but also contribute to clinical symptoms, such as fever and malaise.^269,270^ Following this, the adaptive immune response is activated, characterized by the production of malaria-specific antibodies targeting parasite proteins.^271^ CD8⁺ T cells have been reported to eliminate parasite-infected hepatocytes,^272,273^ whereas CD4⁺ T-cell-dependent antibodies prevent sporozoite invasion of hepatocytes.^274^ During intraerythrocytic development, CD4^+^ T helper cells and potentially γδ T cells exert antiparasitic effects (Fig. 9).^275^ However, our recent study revealed increased expression of host SOD3, which is bound to T cells and is negatively associated with host immunity to malaria.^276^ T cells also play a crucial role in supporting B-cell-mediated antibody production.^277^ However, the high variability of *Plasmodium* antigens and the parasite’s ability to suppress certain immune functions pose significant challenges for the development of an effective immune response in the host.^278^ Recently, the immune landscape established via scRNA-seq revealed that, during *P. falciparum* infection, the proportions of immunosuppressive monocytes, IL-10-producing Tr1 CD4 T cells and IL-10-producing regulatory B cells increased, and tolerogenic markers in natural killer (NK) and γδ T cells were upregulated.^279^

#### CD4^+^ T cells

CD4^+^ T helper (TH) cells, particularly TH1 cells, play an important role in immunity against malaria by producing IFN-γ, which activates macrophages.^280,281^ Both experimental and clinical studies have shown the importance of early IFN-γ production in controlling *Plasmodium* replication,^282,283^ although the precise protective mechanisms are still not fully understood. IFN-γ-producing TH1 cells are linked to resistance during liver-stage *Plasmodium* infection.^284,285^ In addition, IFN-γ-expressing CSP-specific TH1 cells reduce parasite burdens.^286^ However, CD4^+^ T-cell responses can also impair humoral immunity and expand self-reactive B cells.^287^ Within the first four days of infection, a dominant and phenotypically stable CXCR5^+^ TFH population emerges, resulting in a persistent CXCR5^+^ CCR7^+^ TFH/central memory T-cell response. Notably, CD4^+^ T-cell priming by B cells is both essential and sufficient for the establishment of this TFH-dominant response. TH2 cells, characterized by GATA3 and IL-4 production, play a limited role in malaria but are essential for robust CD8^+^ T-cell responses through IL-4-mediated CD4/CD8 cross-talk.^288^ CD8^+^ T-cell activity is significantly diminished without CD4^+^ T-cell support, highlighting their synergy in generating effector cells during immunization with radiation-attenuated sporozoites. Memory CD8^+^ T-cell populations are particularly dependent on CD4^+^ T-cell assistance to control liver-stage parasites.^289^

T follicular helper (TFH) cells, marked by BCL-6, CXCR5, and PD-1 expression, are critical for antibody production and the generation of long-lived plasma cells and memory B cells during *Plasmodium* infection.^290,291^ TFH and TH1 differentiation pathways diverge early in blood-stage infection, influenced by inflammatory monocytes and galectin-1.^292^ Despite this, IL-21 from IFN-γ^+^ TFH cells is crucial for resolving *P. chabaudi* infections by promoting specific IgG responses and immunity to reinfection.^293^

Regulatory T (Treg) cells, characterized by FOXP3 expression, modulate immune responses in malaria. In high-transmission areas, individuals show increased proportions of CD4^+^FOXP3^+^CD127^lo/−^ Tregs with an effector memory phenotype that suppress malaria antigen-induced cytokine production, maintaining immune homeostasis.^294^ Acute infections with *P. vivax* and *P. falciparum* induce expanded Treg populations and altered dendritic cell ratios, correlating with parasite load but not clinical severity.^295^ Increased Treg numbers are also associated with lethal *P. berghei* and *P. yoelii* infections.^296^

#### CD8^+^ T cells

CD8^+^ T cells play a critical role in recognizing pathogen-derived peptides presented by MHC class I molecules on APCs or infected cells, contributing to the clearance of intracellular pathogens and the development of immune memory.^277^ Malaria-specific CD8^+^ T cells have been identified in endemic populations and vaccinated individuals,^297–301^ with the HLA-B^*^53:01 and HLA-C^*^06:02, that were associated with a higher prevalence of *P. falciparum* infection.^302^ Studies in rodent models further corroborate CD8^+^ T-cell-mediated protection, particularly after immunization with irradiated sporozoites.^303^ These cells target sporozoites, liver-stage, and blood-stage antigens of *Plasmodium*, though their role in primary malaria infection remains contentious due to limited hepatocyte infection and a narrow response window.^304–311^ Vaccines that elicit robust CD8^+^ T-cell responses, such as the PfSPZ vaccine, prevent malaria progression and establish long-lived tissue-resident T cells in the liver, underscoring their importance in durable immunity.^308,312^ Attenuated malaria sporozoite vaccines induce protective CD8^+^ T cells in primates, as demonstrated by the finding that CD8^+^ T-cell depletion via cM-T807 leads to malaria infection in previously protected monkeys, whereas those with intact CD8^+^ T cells remain protected.^309^ Although radiation-attenuated sporozoite (RAS) immunization can generate high proportions of CD8 + T cells, this may still not be sufficient for establishing sterile immunity, emphasizing the complex role of CD8^+^ T-cell responses in malaria vaccine efficacy.^311^

CD11c^+^ dendritic cells play a key role in priming CD8^+^ T cells against pre-erythrocytic parasites via cross-presentation of sporozoite antigens in skin-draining lymph nodes.^311,313,314^ Immunization with irradiated sporozoites induces robust protective CD8^+^ T-cell responses, with dendritic cells in cutaneous lymph nodes initiating these responses after mosquito bites. Once activated, CD8^+^ T cells migrate to systemic sites, such as the liver, in an S1P-dependent manner and subsequently recognize antigens on hepatocytes rather than relying on bone marrow-derived antigen-presenting cells.^314^ Another study revealed that sporozoites are directly taken up by lymph node-resident CD8α^+^ dendritic cells, which then form clusters with CD8^+^ T cells, facilitating antigen presentation and priming.^315^ However, genetically attenuated parasites that are arrested in the late liver stage elicit stronger CD8^+^ T-cell responses than those arrested earlier.^316^ Live attenuated vaccines generate robust CD8^+^ T-cell-mediated immunity, but the precise dynamics of CD8^+^ T-cell priming in natural infections or vaccination contexts remain an area of active investigation. Immunization with genetically attenuated *P. berghei* sporozoites lacking the microneme protein P36p provides extended protection lasting 12 to 18 months in mice, with efficacy maintained even with reduced dosages and alternative routes of administration.^317^ CD8^+^ T-cell responses may be primed not only in liver-draining lymph nodes but also in the spleen,^318^ with the generation and maintenance of these responses influenced by additional immune cells such as NK cells, helper T cells, and regulatory T cells, underscoring the need for a deeper understanding of these dynamics to develop strategies for robust and enduring immunity against malaria.^318–321^

CD8^+^ T cells may contribute to the pathogenesis of CM,^322^ a severe complication of malaria, by targeting infected reticulocytes and endothelial cells, leading to BBB disruption.^323–325^ H-2Kb and H-2Db class I molecules on brain endothelial cells uniquely influence disease progression, CD8 + T-cell activation, and BBB disruption; their ablation significantly mitigates ECM pathology and preserves BBB integrity.^326^ scRNA-seq revealed extensive infiltration and high activation of CD8^+^ T cells in the brainstem during ECM, with a subset of Ki-67^+^ CD8^+^ T cells exhibiting elevated levels of activation- and proliferation-related genes, suggesting antigen exposure by brain parenchyma cells; these CD8^+^ T cells were the sole source of IFN-γ, and their activity was modulated by astrocyte-mediated cross-presentation and upregulation of the immune checkpoint molecules PD-1 and PD-L1.^327^ Further research is needed to understand the full scope of the functions of CD8^+^ T cells and their potential in the development of effective malaria vaccines and treatments.

Memory CD8^+^ T-cell-mediated immunity against liver-stage *Plasmodium* infection involves IFN-γ and TNF-α as crucial noncytolytic factors, with perforin playing a species-specific role. While IFN-γ is essential for protection against both *P. berghei* and *P. yoelii*, perforin is critical only for *P. yoelii*, and TNF-alpha neutralization significantly impairs memory CD8^+^ T-cell-mediated protection across both parasite species.^328^ Consistent with the above findings, natural and recombinant human interferons, particularly Hu IFN-γ, effectively inhibited hepatic schizogony of *P. falciparum* at low concentrations, with postinoculation application showing significant inhibitory effects beyond parasitostasis, whereas Hu IFN-α, -β, and IL-1 also had inhibitory effects but at relatively high concentrations or when administered prior to inoculation.^329^ Compared with other tissues, effector memory CD8^+^ T cells rapidly infiltrate the liver within 6 h of malaria infection, mediating pathogen clearance through LFA-1 and liver phagocyte-dependent mechanisms, with a shorter recruitment time (within 6 h) compared to other cells.^330^ Interestingly, CD8^+^ T cells expressing inhibitory molecules such as PD-1 and LAG-3 exhibit suppressive, rather than exhausted, features.^331^

CD8^+^ T cells are integral to malaria immunity, particularly in vaccine-induced protection and liver-stage infection control. However, their role in primary infection and pathogenesis, especially in CM, underscores their complexity. Further research is essential to fully elucidate their functions and optimize strategies for malaria vaccine development.

#### γδ T cells

γδ T cells are a subgroup of T cells characterized by distinct TCRγ and TCRδ chains, accounting for approximately 4% of all T cells in healthy adults.^332–335^ Their contributions to host immunity are complex and varied due to their wide range of effector functions, which are influenced by tissue microenvironments.^335^ In malaria, the role of γδ T cells, particularly those expressing Vγ9^+^Vδ2^+^ chains, remains poorly understood. These cells expand during primary *P. falciparum*^336,337^ infections and are correlated with protection.^337^ Studies in endemic regions indicate that recurrent malaria challenges might influence γδ T-cell expansion, potentially aiding in clinical malaria control as individuals age.^338,339^ In African children with *P. falciparum* malaria, the majority of the perturbing γδT cells expressed V delta 1 and exhibited a highly activated phenotype, with TCR analysis revealing that the expanded V delta 1^+^ population was highly polyclonal, used various V gamma chains, and predominantly produced IFN-g, although fewer V delta 1^+^ T cells produced TNF-α than the overall CD3^+^ T-cell population.^336^ Interestingly, Vγ9^+^Vδ2^+^ γδ T cells expand during acute infections but tend to contract with subsequent exposures, despite reactivating each time.^337,338,340^ Recently, scRNA sequencing revealed an increase in immunosuppressive monocytes and the upregulation of tolerogenic markers in NK and γδ T cells.^279^ And placental *P. falciparum* infection represented altered γδ T-cell proportions, with increased Vδ1^+^ subsets and decreased Vδ2^+^ proportions. These changes, along with altered activation and exhaustion in marker expression, correlate negatively with maternal hemoglobin levels and birth weight.^341^

In rodent malaria models, γδ T cells expand clonally during the blood stage and support TFH cell responses by producing IL-21. They help to control recrudescence via TCR-dependent mechanisms, potentially involving M-CSF production. Their presence correlates with the efficacy of RAS vaccines, as their depletion impairs CD11c^+^ DC influx into the liver and hinders optimal CD8^+^ T-cell responses, reducing sterile immunity.^342–344^ In studies with a murine model, separating liver and blood stages of infection, it was revealed that liver stage-dependent activation of Vγ4^+^ γδ T cells was crucial for mouse survival. Whereas blood-stage parasite loads were associated with cytokine profiles, where low parasite loads promoted IL-17-producing γδ T cells. These cells drive extramedullary erythropoiesis and reticulocytosis, protecting mice from ECM. This protection can be replicated through adoptive transfer of erythroid precursors.^339^

#### Humoral immunity and malaria vaccines

Humoral immunity, which is mediated by antibodies, is crucial in controlling *Plasmodium* infections and mitigating malaria severity.^345^ Antibodies target various antigens of parasites in different life cycle stages, particularly blood-stage antigens such as PfEMP1,^250,346^ MSP1,^347,348^ and circumsporozoite protein (CSP).^349–351^ These antibodies facilitate parasite clearance through mechanisms such as opsonization,^352^ neutralization,^353^ and complement activation.^354,355^ However, naturally acquired humoral immunity against malaria tends to be inefficient and short-lived due to parasite antigenic variation and immune evasion strategies.^356^ Recently, immunization with the single-component SBD1 immunogen, which retains the structure of the AMA1-RON2L complex, was found to elicit more potent strain-transcending neutralizing antibody responses against *P. falciparum* than did immunization with the AMA1 or AMA1-RON2L complex alone, highlighting its potential for advancing malaria vaccine development.^357^

*Plasmodium* infections induce robust B-cell responses,^358,359^ but the maintenance of these responses is hindered by factors such as the parasite-derived metabolic product, hemozoin, which activates inflammasomes and restricts long-term antibody production and memory B-cell formation.^360^ The latest results from our laboratory show that B-cell differentiation into IL-35^+^ Bregs during *Plasmodium* infection, driven by TLR9 activation and distinct signaling via IRF3 pathways, plays a critical role in malaria pathology, with IL-35^+^ Bregs contributing to the development of ECM and influencing parasitemia levels. The generation of durable immunity is further complicated by the need for continuous exposure to the parasite to maintain antibody levels, as well as the parasite’s ability to undergo antigenic variation, which challenges the immune system’s capacity to form effective memory responses. During malaria infection, the rapid development of short-lived plasmablasts disrupts the formation of long-lasting humoral immunity by impairing germinal center responses, as these plasmablasts exhibit metabolic hyperactivity that deprives the germinal center of necessary nutrients.^361^ However, therapeutic interventions targeting metabolic constraints can enhance parasite clearance and promote the development of protective immune memory. Additionally, cytokines such as GM-CSF and IL-3, produced by IgM^+^ and IgG^+^ B1b B cell plasmablasts, play an important role in the immune response.^362^ Early in the infection, these cytokines are primarily produced by IgM+ B1b B cells, with a later shift to IgG^+^ plasmablasts, suggesting an isotype switch and highlighting the functional plasticity and phenotypic heterogeneity of innate B1 B cell subsets.^362^

Current malaria vaccines aim to elicit strong humoral and cellular immune responses (Table 2).^363^ The RTS,S/AS01_E_ (Mosquirix) vaccine, which targets the CSP, is the most advanced malaria vaccine and has been approved for use in endemic regions.^364^ RTS,S/AS01_E_ primarily induces antibody and CD4^+^ T-cell responses that target preerythrocytic-stage parasites.^365^ Despite its limited efficacy, studies have shown that delayed fractional dosing of RTS,S/AS01_E_ can enhance the quality and longevity of the humoral response by promoting a balanced production of polyfunctional antibodies against CSP and Pf16 antigens.^366^ Antibody responses to a three-dose primary vaccination series were significantly greater observed in Ghana than in Malawi and Gabon. However, neither antibody levels nor vaccine efficacy against initial malaria cases were influenced by background incidence or parasitemia during the vaccination series.^367^ A phase 1 clinical trial demonstrated that the combination of full-length *P. falciparum* MSP1 with the GLA-SE adjuvant is safe, well tolerated, and immunogenic, inducing lasting MSP1-specific IgG and IgM responses and memory T-cell responses, making it a promising candidate for further efficacy evaluation in malaria vaccine development (EudraCT 2016-002463-33).^368^ AMA1 has been identified as a conserved and essential malaria vaccine target. A human monoclonal antibody targeting AMA1 domain II, which effectively inhibits *P. falciparum* growth through a novel mechanism independent of RON2 binding, was successfully isolated and optimized, demonstrating the potential of phage display libraries for developing potent blood-stage malaria interventions.^369^ Additionally, a plant-based vaccine incorporating the AMA1 and MSP1_19_ proteins induced specific immune responses in test animals, showing promise as a subunit vaccine.^370^ Compared with vaccines targeting the F2 domain and full region II, vaccines targeting the EBA-140 F1 domain, which includes the crucial SA-binding pocket, present significantly better parasite neutralization, highlighting the importance of targeting functionally relevant epitopes for enhancing malaria vaccine efficacy.^371^

**Table 2:** Candidate malaria vaccines in clinical development

Stage	Phase 1	Phase 2	Phase 3
Pre-erythrocytic	BNT165b1 (CSP mRNA) rCSP/AP10-602 (FL-CSP) FMPO13/ALFQ (FL-CSP) FMP014/ALFQ (CSP, nanoparticles) VLPM01 (CSP, virus-like particle) DNA-ChAd63 *Pf*CSP (prime-boost) DNA-ChAd63 *Pf*CSP *Pf*AMA1 ME-TRAP (prime-boost) PfGAP3-KO (genetically attenuated sporozoite) PfSPZ-GA1 (genetically attenuated sporozoite)	PfSPZ (Radiation-attenuated sporozoite)	R21/MatrixM
Blood Stage	BK-SE36/CpG (PfSERA5 antigen) *Pf*7G8 (Chemical attenuated parasite) PRIMVAC (targeting VAR2CSA, for Malaria in Pregnancy) PAMVAC (targeting VAR2CSA, for Malaria in Pregnancy)	Rh5.1/AS01 (invasion molecule) ChAd63-MVARh5 (invasion molecule) RH5.1/Matrix-M	
Sexual Stage	*Pf*s25-IMX313/MatrixM (zvgote/ookinete) *Pf*s25M-EPA/AS01B (zvgote/ookinete) *Pf*s230D1-EPA/MatrixM (gamete surface antigen) R0.6 C(*Pf*s48/45) (gamete surface antigen) AnAPN1/GLA-LSQ (mosquitos midgut antigen)	Pfs230D1M-EPA/AS01B (gamete surface antigen)	
Multistage	RH5.2-VLP plus R21 in Matrix-M (planning)		

The data were from the WHO website (https://www.who.int/data/gho)

The two pre-erythrocytic vaccines, R21/Matrix-M and RTS,S/AS01, do not elicit protection against blood-stage parasites. Rh5.1/Matrix-M is a blood-stage *P. falciparum* vaccine candidate. In a phase 1b trial, the RH5.1/Matrix-M malaria vaccine candidate exhibited good safety and immunogenicity in both adults and children in a malaria-endemic area, with sera from all children in the delayed third-dose regimen displayed a growth inhibition activity (GIA) previously linked to protective immunity. The vaccine induced strong anti-RH5.1 antibody responses and showed promising results for further efficacy trials against clinical malaria in young African children.^372^ In a phase 2b trial, the RH5.1/Matrix-M vaccine also demonstrated good safety and immunogenicity in Burkinabe children, with a 55% vaccine efficacy in the delayed third-dose regimen, alongside strong antibody responses and significant *P. falciparum* growth inhibition activity in vitro.^373^ A recent study investigated the potential of enhanced vaccine efficacy by immunization with a cocktail of the RCR-complex, consisting of RH5, CyRPA, and RIPR, compared to RH5 alone. Despite the identification of additive or synergistic effects of monoclonal antibodies targeting different antigens, vaccination with the RCR-complex in rats did not outperform RH5 alone due to RIPR immuno-dominance; however, combining RH5 with a fusion protein (R78C) improved parasite growth inhibition, supporting the advancement of the RH5.1 + R78C/Matrix-M™ vaccine to clinical trials.^374^

Another promising approach is to block malaria transmission by targeting antigens expressed during the mosquito stage of the parasites, such as the AnAPN1 vaccine.^375^ This vaccine induces functional antibodies that prevent the development of the parasite within the mosquito vector, thus curbing transmission.^375^ Immunology-based strategies have been employed to increase the efficacy of AnAPN1, resulting in more potent and durable antibody responses.^375^ Ongoing studies are aiming to develop vaccines that not only provide short-term protection but also induce long-lasting immunity.

Vaccine studies on *P. vivax* and other malarial species are also progressing. A study characterized the sequence and structural diversity of P. vivax merozoite surface protein 3 (PvMSP3γ) by analyzing 118 complete *pvmsp3γ* sequences from Thailand and 9 reported sequences, revealed 86 distinct haplotypes. The findings suggest that polymorphism in PvMSP3γ is driven by recombination and natural selection, with structural variations potentially complicating vaccine development due to alterations in immunogenic epitopes among variants.^376^ Another study analyzed natural IgG antibody responses in 246 symptomatic *P. vivax* malaria patients to PvMSP3γ recombinant proteins revealed widespread seropositivity and a strong correlation with previous malaria episodes. The findings highlighted the presence of B-cell epitopes across PvMSP3γ, with predominant IgG1 and IgG3 responses.^377^ Moreover, other studies investigated the immunogenicity of *P. ovale* merozoite surface protein 4 (PoMSP4), a potential vaccine candidate. The findings exhibited that both *P. ovale curtisi* (PocMSP4) and *P. ovale wallikeri* (PowMSP4) protein sequences are highly conserved, and the recombinant proteins could induce strong humoral and cellular immune responses in mice, including high antibody titers and significant splenocyte proliferation, suggesting its potential as a vaccine target for malaria.^378^

Naturally, acquired immunity to malaria develops gradually after repeated exposure to the parasites, leading to the accumulation of antibodies that target various *Plasmodium* antigens.^356^ Studies in endemic regions have shown that individuals frequently infected with malaria parasites eventually develop a repertoire of antibodies that can confer partial protection against clinical malaria.^356,379^ However, this immunity is often incomplete and can wane in the absence of continuous exposure.^380^ Children in high-transmission areas are at greater risk because of their less mature immune systems and lower antibody titers.^381^ Protective immunity requires a threshold concentration of antibodies against merozoite antigens, such as MSP1 and AMA1, which are crucial for inhibiting parasite invasion of red blood cells.^381^ Recently, the development of an engineered SpyCatcher-mi3 nanoparticle has shown significant potential in the field of nanobiotechnology and vaccine development. This nanoparticle efficiently binds to malaria antigens, eliciting strong immune responses and demonstrating its versatility as a future platform for medical advancements.^382^

Understanding the dynamics of naturally acquired immunity to malaria will provide valuable insights for vaccine development. For example, insights into how the immune system responds to repeated infections and how memory B cells are generated and maintained can facilitate the design of vaccines that mimic natural exposure and increase long-term immunity.^383^ Additionally, the identification of correlates of protection, such as specific antibody profiles that confer immunity, is essential for evaluating vaccine efficacy and guiding the development of more effective immunization strategies.^384^

In conclusion, while significant progress has been made in understanding the mechanisms of humoral and cellular immunity and the development of malaria vaccines, challenges remain in achieving durable and broadly protective immunity. Continued research into the molecular and cellular pathways involved in immune responses to *Plasmodium* infection, as well as innovative vaccine strategies, will be critical in the global effort to control and eventually eradicate malaria.

### Therapeutic targets of anti-malaria drugs and progress in clinical application

**Fig. 10:**
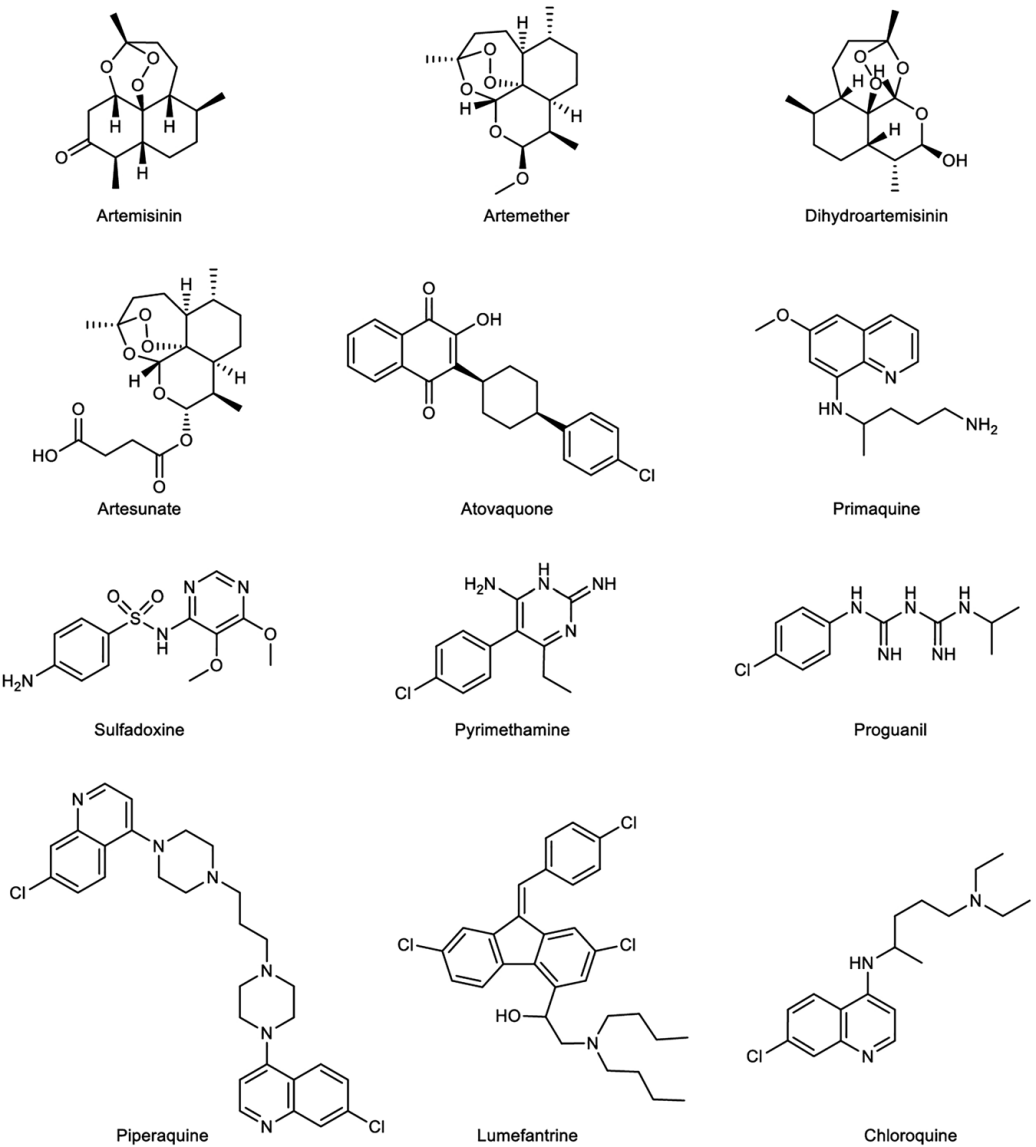
The drugs and structures of anti-malaria drugs were approved by FDA. The drugs and structures shown include well-known treatments such as Artemisinin, Artemether, Dihydroartemisinin, Artesunate, and others like Atovaquone, Pyrimethamine, and Chloroquine, which are currently used in the treatment of malaria

Malaria remains a significant global health challenge, necessitating ongoing research on therapeutic targets and clinical interventions. This section is divided into three parts. The first part provides information on malaria drugs currently approved by the Food and Drug Administration (FDA) (Fig. 10). The second part discusses frontline antimalarial treatments and their mechanisms of resistance (Fig. 10). The third part summarizes the conditions and objectives that must be met in the development of new drugs to counteract resistance and the new drug list in malaria treatment.

Malaria can be effectively treated when therapy is initiated promptly, but delayed treatment can lead to severe or even fatal outcomes.^105,385^ The choice of treatment depends on several factors, including the parasite species, the severity of the infection, the risk of drug resistance based on the region where the infection was contracted,^386^ as well as the patient’s age,^387^ pregnancy status,^388^ or breastfeeding considerations.^389^ Current FDA-approved malaria treatments, including Artemether-Lumefantrine (Coartem^®^),^390^ Atovaquone-Proguanil (Malarone™),^391^ and Primaquine,^392^ play crucial roles in combating malaria but face notable limitations in clinical settings. Artemether-Lumefantrine is widely used for uncomplicated *P. falciparum* malaria and is effective even during pregnancy^393,394^; however, it requires precise dosing schedules and administration with food to optimize absorption,^395,396^ which can be challenging in resource-limited or emergency contexts. Atovaquone-Proguanil, favored for its shorter treatment duration and ease of administration,^397,398^ is not recommended during pregnancy,^399^ in infants weighing less than 5 kg,^400^ or for breastfeeding mothers of such infants,^401^ restricting its use in some vulnerable populations. Primaquine is useful for eliminating *P. vivax* and *P. ovale* hypnozoites to prevent relapses^106^ but poses significant risks of hemolytic anemia in individuals with glucose-6-phosphate dehydrogenase (G6PD) deficiency.^402^ This necessitates quantitative G6PD testing before administration, a resource often unavailable in endemic areas, further complicating its deployment. Additionally, Primaquine is contraindicated during pregnancy and also poses risks for breastfeeding infants without confirmed normal G6PD activity.^403^ Drug resistance further confounds these issues, as resistance to chloroquine and other classical antimalarials has necessitated the adoption of combination therapies, which, while being effective, increase costs^404^ and logistical complexities. Other challenges include ensuring safe and effective treatment for pregnant women and children, both are highly vulnerable groups, as well as achieving patient adherence to the often complex and prolonged treatment regimens.

**Fig. 11:**
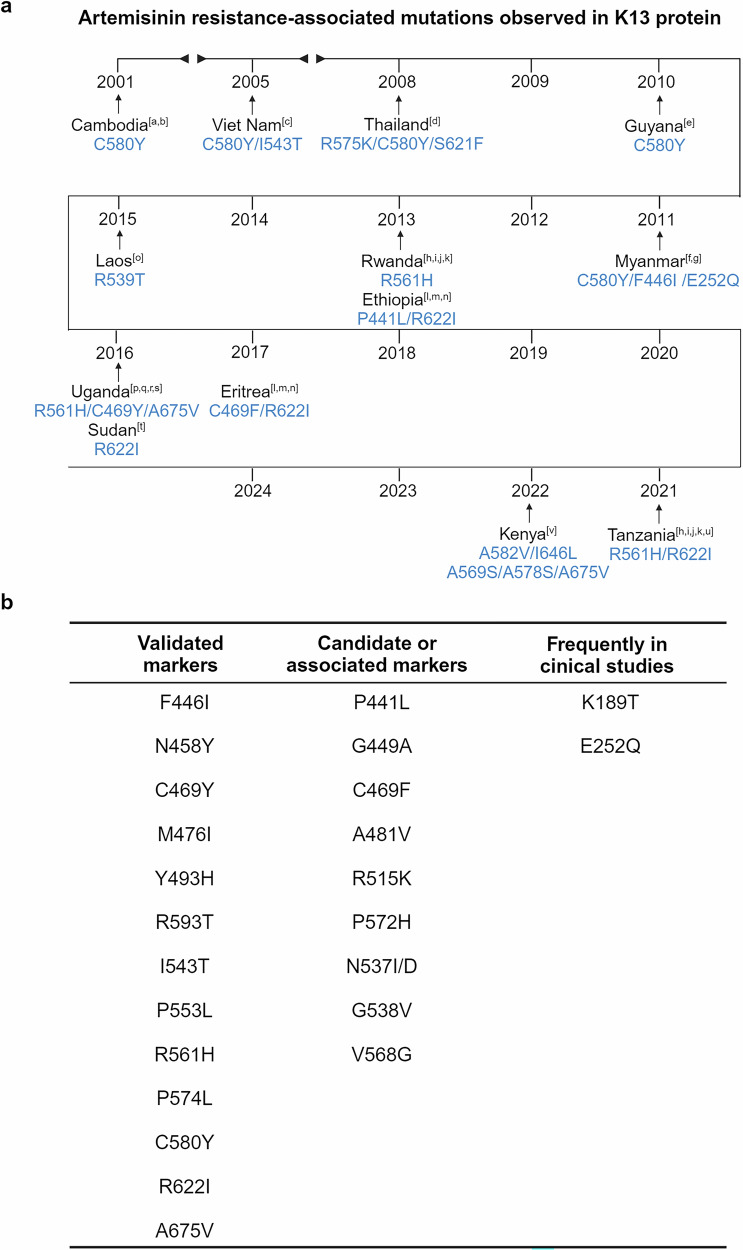
PfKelch13 mutations. **a** The global emergence of ART resistance-associated mutations in the *P. falciparum* K13 protein from 2001 to 2024.The references are shown in [a],^67^ [b],^510^ [c],^511^ [d],^512^ [e],^513^ [f],^514^ [g],^415^ [h],^515^ [i],^516^ [j],^517^ [k],^518^ [l],^519^ [m],^520^ [n],^521^ [o],^522^ [p],^523^ [q],^524^ [r],^525^ [s],^416^ [t],^526^ [u],^527^ [v].^528^ **b** Markers of artemisinin resistance in pfkelch13 and commonly observed mutations in clinical studies. The data was from WHO (https://www.who.int/news-room/questions-and-answers/item/artemisinin-resistance). This figure was created with BioRender.com

The genetic variability of the human malarial parasite *P. falciparum* is suggested to be the main cause of its resistance to drug treatments.^405^ The rapid evolution of *P. falciparum* genomes in response to drug pressure necessitates the continuous monitoring and updating of antimalarial strategies.^406^ Frontline antimalarial therapeutics primarily encompass ART and its derivatives, chloroquine, and sulfadoxine–pyrimethamine.^407^ Consequently, this paragraph will systematically review drug resistance according to the aforementioned drug classes. ART and its derivatives are most effective against *Plasmodium* species, but their efficacy is threatened by the emergence of ART-resistant parasites.^408^ Clinically, partial resistance to ART is characterized by a delayed clearance phenomenon, with a parasite clearance half-life >5 h^409^ or parasites persist up to a standard 3-day ACT treatment regimen. However, by day 28, the clinical and parasitic response rates remain unaffected if the partner drug retains its efficacy.^410^ This suggests that initial delays in parasite clearance do not necessarily indicate treatment failure.^410^ In *P. falciparum*, resistance to ART is often quantified in vitro as more than 1% of early ring-stage parasites surviving a 6-h exposure to 700 nM ART derivative dihydroartemisinin (DHA).^411^ *P. falciparum* partial resistance mechanisms to ART and DHA have been suggested to primarily involve genetic mutations in the *P. falciparum* kelch 13 (PfK13), alterations in parasite metabolic pathways, and epitranscriptomic and epigenetic mechanisms. Consequently, this paragraph will systematically explore resistance to ART and DHA through these aforementioned mechanisms. *Pf*K13 is a protein encoded by the *kelch13* gene,^412^ which is involved in hemoglobin trafficking by parasites during the asexual blood stage.^412^ Mutations in PfK13, including Y493H, R539T, I543T, and C580Y mutations (Fig. 11a, b), have been linked to delayed parasite clearance following ART treatment.^413,414^ C580Y mutation in the PfK13 protein has become dominant in parasite strains common in regions such as the eastern Greater Mekong Subregion.^415^ This mutation spreads in specific parasite sublineages that may also possess secondary factors that increase resistance or mitigate the adverse effects of mutations on parasite physiology.^415^ While there is a possibility that the C580Y mutation can enhance parasite transmissibility, further research is needed to verify this phenomenon. There are significant differences in the resistance conferred by PfK13 mutations in African isolates,^416^ with some mutations resulting in little to no resistance in vitro.^417^ The competitive fitness of these mutated strains against wild-type strains has also been questioned, and the slower spread of ART-resistant strains in Africa may be due to various ecological and biological factors.^416^

Upon ART treatment, parasites exhibit various responses, including activation of the unfolded protein response, altered mitochondrial physiology, and changes in developmental stages that contribute to resistance.^418–423^ One mechanism underlying ART resistance associated with mutations in the *kelch13* gene involves a reduction in hemoglobin endocytosis, which subsequently leads to decreased levels of Fe^2+^ heme. This reduction is believed to be critical for the activation of ART through the cleavage of its endoperoxide bridge.^424^ Another mechanism is that *PfK13* mutations (Y493H and 580Y) may be associated with alterations in parasite metabolic pathways in ring stage, including the tricarboxylic acid cycle, glycolysis, and amino acid metabolism, in response to DHA exposure.^425^ Furthermore, an epitranscriptomic mechanism involving tRNA hypomodification and codon-biased translation, particularly the modification of mcm⁵s²U on tRNA resulting in tRNA s²U reprogramming, may also regulate PfK13 function to enabled the survival of parasites under ART-induced stress.^426^ Moreover, emerging evidence suggests that multicopy Pfpm2 may compensate for the fitness impacts of various PfCRT isoforms by increasing hemoglobin degradation, potentially contributing to DHA + piperaquine (PPQ) resistance in *P. falciparum*.^427^ The evidence of epidemiology is that following the introduction of DHA-piperaquine in 2010, newly emerged PfCRT mutations rapidly increased in prevalence, reaching more than 98% by 2017 in northern Cambodia. In contrast, after artesunate-mefloquine treatment, the prevalence of parasites with increased copy numbers of *plasmepsin II* (pfpm2) decreased, with nearly half of the piperaquine-resistant strains carrying a single copy of *pfpm2*.^428^ Additionally, a recent study revealed that brief exposure to other ART derivative artesunate (AS) stimulates rosette formation in *P. falciparum*, especially in ART-resistant isolates, enabling infected erythrocytes to survive drug exposure and evade phagocytosis, indicating that AS-mediated rosette formation in late-stage parasites contributes to ART resistance by allowing parasites to persist in less drug-susceptible environments.^429^ Overall, the mechanisms underlying resistance to ART predominantly encompass alterations in heme uptake, metabolic adaptations, epigenetic modifications, and rosette formation.

Chloroquine (CQ) was once an effective antimalarial drug, but resistance mediated by mutations in the *P. falciparum* CQ resistance transporter (PfCRT) has significantly reduced its efficacy against *P. falciparum*. PfCRT is a protein encoded by the *pfcrt* gene in *P. falciparum*.^430^ This protein is located in the digestive vacuole membrane of the parasite.^430^ Mutations in PfCRT lead to a reduction in drug accumulation within the parasite’s digestive vacuole, which is essential for its antimalarial effect.^431^ To date, no fewer than 30 variant residues in PfCRT have been identified, rendering PfCRT an extraordinarily polymorphic protein. Notably, in all resistant parasites, lysine 76 (K76) in PfCRT is replaced with an uncharged amino acid, either a threonine (76T) in the case of virtually all field isolates (with one reported exception of a 76A variant)^432^ or an asparagine or isoleucine (76 N/I) in laboratory-adapted lines exposed to CQ.^433^ The 3.2 Å resolution structure of the PfCRT protein from CQ-resistant but PPQ-sensitive *P. falciparum* 7G8 parasites revealed that mutations contributing to resistance occur in different helices lining a central negatively charged cavity-the primary interaction site for the positively charged drugs. Functional analyses demonstrated that the newly emerging PfCRT mutations, namely F145I and C350R, enable PPQ transport and confer resistance, providing insights into the distinct mechanisms by which PfCRT mediates CQ and piperaquine resistance.^434^ Overall, these results indicate that drug resistance is an ongoing battle. *P. falciparum* can utilize gene mutations or amplifications that confer resistance to other antimalarial drugs to resist new antimalarial medications. Furthermore, another vacuolar protein, the amino acid transporter (PfAAT1), was also found to be associated with CQ resistance. This may be a compensatory evolution in *P. falciparum*. A longitudinal genomic analysis of Gambian *P. falciparum* isolates revealed the PfAAT1 variant S258L, which increased in frequency alongside the PfCRT K76T mutation, and gene editing confirmed that this variant enhances CQ resistance at the cost of fitness, with other regional variants, such as F313S, mitigate resistance while restoring fitness.^435^ Moreover, the nondrug-related function of PfCRT in *P. falciparum* was determined by generating a conditional knockdown mutant, revealing its potential role in oligopeptide transport.

SP is used throughout Africa for intermittent preventive treatment of malaria, but parasite resistance to SP threatens its efficacy. PfDHFR (*P. falciparum* dihydrofolate reductase) and PfDHPS (*P. falciparum* dihydropteroate synthetase) are functionally critical enzymes in *P. falciparum* that are linked to SP resistance.^436^ Mutations in the *PfDHFR* (N51I, C59R, S108N, and I164L)^437,438^ and *PfDHPS* (I431V, S436 A/F, A437G, K540 E/N, A581G, and A613T)^439,440^ genes are key contributors to SP resistance. The combination of triple pfdhfr (N51I/C59R/S108N) and double pfdhps (A437G/K540E) mutations is a strong predictor of SP treatment failure and reduces the efficacy of SP-based interventions, especially in areas where the prevalence of dhps K540E exceeds 50%.^441,442^ In 2019, a study conducted on Bioko Island revealed a high prevalence of these mutations, underscoring the necessity for ongoing molecular monitoring and control efforts to manage SP resistance effectively.^443^ The drug CID 10476801 has emerged as a potent inhibitor in docking studies of pyrimethamine derivatives, suggesting another potential avenue for treatment.^444^ Further research using pharmacophore modeling and docking has identified several natural products as potential PfDHFR inhibitors.^445^ These candidates show promise for development against both WT and mutant PfDHFR strains, which could lead to new, effective treatments for malaria.^445^

The criteria for substitution of existing therapies with novel treatments are rigorously defined. Moreover, the development of new treatments for severe malaria remains necessary, especially in cases where oral medications are not suitable. Additional efforts are also required to create drugs capable of managing asymptomatic infections and eliminating dormant parasites in *P. vivax* malaria. The second target focuses on chemoprevention and prophylaxis, driven by the absence of a fully protective vaccine. Chemoprevention involves the administration of a full treatment dose to individuals in highly endemic areas to control transmission, as some individuals may be asymptomatic carriers. Prophylaxis entails medication for individuals who are at risk of infection. The third objective is to develop new antimalarial drugs that encompass several key features. The first is stability, particularly under conditions of high temperature and humidity. The second is the consideration of specific needs for children and pregnant women, including safety and appropriate drug formulation. The third is to ensure that the cost is kept as low as possible. Additionally, in malaria, as with many infectious diseases, drug development is more complex because an optimal medicine is often combined of two or more active drugs.^410^

**Fig. 12:**
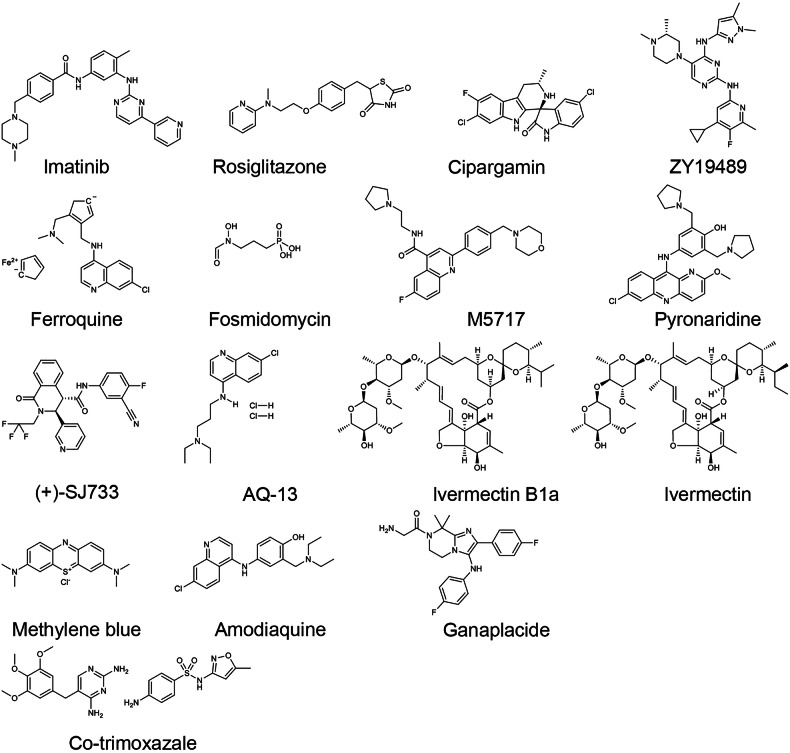
The structures of new anti-malaria drugs. The drugs presented include novel and experimental compounds that have shown potential against *Plasmodium* infections, such as Imatinib, Rosiglitazone, Cipargamin, ZY19489, and Ferroquine, among others. These structures represent different classes of anti-malaria agents currently under investigation or development, encompassing a range of mechanisms of action aimed at combating malaria

Recent advancements in antimalarial drug development have introduced a diverse array of novel therapeutics and combination therapies aimed at enhancing efficacy, overcoming drug resistance, and improving patient outcomes (Fig. 12). Sevuparin, a modified heparin derivative, disrupts the sequestration of *Plasmodium*-infected erythrocytes, thereby reducing microvascular obstruction in severe malaria cases.^446,447^ Imatinib, originally for cancer treatment, has been repurposed to inhibit essential kinases in the malaria parasite, showing promise in reducing parasitemia.^448–451^ Rosiglitazone, a PPAR-γ agonist, is being evaluated as an adjunctive therapy to improve clinical outcomes in severe malaria.^452–455^ In a phase IIa randomized, double-blind, placebo-controlled trial in Mozambique, adjunctive rosiglitazone was found to be safe and well-tolerated in children with uncomplicated malaria, supporting its further evaluation as an adjunctive therapy for severe malaria.^453^ But in another randomized, double-blind, placebo-controlled trial involving 180 Mozambican children with severe malaria, adjunctive rosiglitazone treatment did not significantly reduce circulating angiopoietin-2 levels or improve clinical outcomes compared to placebo when administered alongside artesunate.^452^ Cipargamin, a spiroindolone compound targeting the PfATP4 protein, disrupts parasite ion homeostasis, leading to rapid clearance.^456–459^ Combination therapies such as ZY19489 (sutidiazine) with ferroquine^410,460^ and Fosmidomycin with piperaquine^461,462^ offer synergistic effects by targeting multiple parasite pathways, enhancing efficacy against resistant strains. M5717 (DDD107498), an inhibitor of the *P. falciparum* translation elongation factor 2, demonstrates potent antimalarial activity in clinical trials.^463–466^ Pyronaridine is under optimization in combination with artesunate to prevent resistance development,^467–470^ while (+)-SJ733, another PfATP4 inhibitor, is undergoing clinical evaluation for its effectiveness in both uncomplicated and severe malaria.^471–474^ AQ-13, a novel quinoline-based antimalarial, shows efficacy against multiple *Plasmodium* species,^475–477^, and L9LS mAb, a monoclonal antibody, aims to neutralize the parasite and prevent its proliferation.^478,479^ New drugs such as Ivermectin (LYN-163)^480–482^ and combination therapies such as methylene blue with amodiaquine leverage unique mechanisms, including redox-active properties and traditional antimalarial action, to enhance parasite killing.^483–487^ Moreover, recent Phase II data indicate that the novel antimalarial ganaplacide is effective and well-tolerated for treating uncomplicated *P. falciparum* malaria in adults, adolescents, and children. Ganaplacide targets the parasite’s internal protein secretory pathway, and its reduced susceptibility is linked to mutations in the *P. falciparum* genes *CARL*, *UDP-galactose*, and *Acetyl-CoA transporters*. When combined with lumefantrine, which inhibits the parasite’s conversion of toxic heme to non-toxic hemozoin, this combination enhances the treatment’s efficacy.^488–491^ Moreover, in a randomized, double-blind, placebo-controlled clinical trial conducted in Gabon and Mozambique, intermittent preventive treatment with DHA-piperaquine for pregnant women with HIV receiving co-trimoxazole prophylaxis was found to be safe and effective, significantly reducing the incidence of clinical malaria and overall *P. falciparum* infection.^492^ These innovative approaches collectively represent a comprehensive strategy to combat malaria, addressing critical challenges such as drug resistance and treatment efficacy, and hold significant promise for advancing global malaria control and eradication efforts.

These findings underscore that drug resistance in *P. falciparum* is an ongoing battle, as the parasite continuously evolves through gene mutations and amplifications to resist new antimalarial medications. Future efforts must focus on enhanced surveillance, a deeper understanding of resistance mechanisms, the development of new drugs effective against resistant strains, the optimization of combination therapies, and global collaboration to adapt to the evolving challenge posed by *P. falciparum*.

### Conclusion and future perspectives

Malaria, caused by *Plasmodium* parasites transmitted by Anopheles mosquitoes, continues to be a major global health challenge, particularly in sub-Saharan Africa, where most malaria cases and malaria-related deaths occur. The life cycle of *Plasmodium* involves complex interactions between the parasite and its mosquito and vertebrate hosts. The parasite undergoes various developmental stages, including the liver and blood stages, each of which contributes to disease pathogenesis and transmission dynamics. The emergence of drug-resistant strains as a result of the genetic mutagenesis of *P. falciparum* poses challenges in treatment and disease control. Mutations in genes such as PfK13 (associated with ART resistance) and PfCRT (associated with CQ resistance) highlight the need for ongoing surveillance and the development of new therapeutic strategies.

Advances in genomic and molecular biology have provided deeper insights into *Plasmodium* pathogenesis, immune evasion, and parasite‒host interactions. Genomic studies have revealed significant genetic diversity among *Plasmodium* species, influencing parasite evolution, population genetics, and drug resistance mechanisms. ScRNA-seq analyses have further revealed the transcriptional landscapes of *Plasmodium* parasites at various stages and in different environmental settings, aiding in the identification of potential targets for vaccine development and therapeutic interventions. Future research should focus on expanding these technologies to unravel the molecular mechanisms underlying parasite ability in adaptation to different environments and evade the host immune system. Additionally, a more comprehensive understanding of gene regulation in both human and rodent species, as well as the role of genomic diversity in drug resistance, will be crucial for developing more effective therapeutic and preventive measures. Moving forward, the integration of cutting-edge technologies, such as scRNA-seq and spatial transcriptomics, with advanced computational tools, will be essential for advancing our knowledge of parasite biology and overcoming the challenges in malaria control.

Host immune responses to malaria are complex and involve both innate and adaptive immunity, which includes the activation of macrophages and dendritic cells and the production of proinflammatory cytokines crucial for controlling parasite replication and clinical symptoms. Adaptive immune responses, particularly the production of specific antibodies and the activation of T cells, play essential roles in long-term immunity and protection against malaria. However, the development of effective and durable immunity is hindered by the parasite’s ability to evade the immune system through antigenic variation and immune suppression mechanisms.

Efforts to develop malaria vaccines have yielded some success, with the RTS,S/AS01_E_ (Mosquirix) vaccine showing certain protection efficiency after several immunization. Ongoing research aims to improve vaccine design and efficacy by targeting multiple stages of the parasite’s life cycle and increasing the quality and longevity of immune responses. Innovative approaches, such as transmission-blocking vaccines, hold promise for advancing malaria control and eradication strategies.

Developing a comprehensive strategy for future malaria research requires focused attention on several critical areas. First, in-depth studies of parasite biology and genetics are essential, including genomic sequencing to identify genetic variations linked to drug resistance and transmission dynamics. Dissection of the molecular mechanisms at each stage of the parasite’s life cycle will help identify potential intervention points. A thorough understanding of the host immune response and genetics is also crucial. This includes exploring how the parasite evades immune detection, identifying host genetic factors influencing susceptibility or resistance, and developing strategies to enhance immune reactivity for more effective parasite clearance.

Vector biology and control are key research areas, particularly the genetic study of *Anopheles* mosquitoes to uncover factors that contribute to vector competence and insecticide resistance. Innovative vector control strategies, such as genetically modified mosquitoes and biological control agents, should also be explored. Additionally, vaccine development through antigen discovery, optimizing antigen delivery platforms, and rigorous clinical trials is critical. Improving diagnostics and surveillance, through the development of rapid diagnostic tests, molecular diagnostic tools, and leveraging geospatial technologies for more effective monitoring, will also be essential components of future malaria research efforts.

The control of malaria requires a comprehensive and integrated approach. Integrated Vector Management combines chemical, biological, and environmental strategies to effectively control mosquito populations while addressing insecticide resistance through measures such as rotating insecticides and incorporating non-chemical controls. Community engagement is crucial for ensuring the sustainability and acceptance of these initiatives. Universal access to effective treatment demands widespread distribution of antimalarial drugs, particularly ACTs, alongside regular updates to treatment guidelines based on evolving resistance patterns. Strengthening health infrastructure is essential for providing timely diagnosis and treatment, especially in remote areas. Preventive measures, including the use of insecticide-treated nets (ITNs), indoor residual spraying (IRS) in high-transmission zones, and chemoprophylaxis for vulnerable populations, are critical to reducing mosquito bites and preventing malaria transmission. Furthermore, enhancing surveillance and response systems through early detection, data integration, and adaptive management allows for rapid identification and containment of outbreaks.

Future drug development may aim to disrupt the malaria life cycle by focusing on both the pathogen and the host, as well as their interactions. Parasite targets include novel enzymes and metabolic pathways unique to *Plasmodium*, such as kinases and energy-associated pathways, to inhibit parasite development and replication. Strategies to block transmission involve targeting gametocyte development and preventing the parasite from invading the mosquito midgut. On the host side, enhancing the immune response to more effectively clear the parasite and reducing immunopathology are key strategies. Targeting host metabolic pathways to deprive the parasite of necessary nutrients or manipulating iron metabolism can inhibit parasite growth. Additionally, disrupting host-pathogen interactions with adhesion inhibition molecules can prevent complications like severe anemia and dysfunction of critical organs. Emphasizing multi-target and combination therapies through polypharmacology and optimized combination regimens can reduce the likelihood of resistance development and improve treatment efficacy. By integrating these approaches, future drug development can achieve more effective and sustainable malaria control.

In conclusion, while significant progress has been made in understanding the molecular and genetic underpinnings of malarial parasites, challenges remain in achieving comprehensive and sustainable disease control. Continued research into parasite biology, host‒parasite interactions, and immune responses, coupled with the development of novel therapeutic and preventive measures, is essential for overcoming the persistent burden of malaria.
